# Detection and characterisation of high pathogenicity avian influenza virus (H5N1/H5N8) clade 2.3.4.4b, Hong Kong SAR, China, 2021 to 2024

**DOI:** 10.2807/1560-7917.ES.2025.30.1.2400839

**Published:** 2025-01-09

**Authors:** Wanying Sun, Ka Tim Choy, Ka Man Cheng, Christopher J Brackman, Samuel MS Cheng, Thomas HC Sit, Anne CN Tse, Leslie D Sims, Haogao Gu, Amy WY Tang, Andrew NC Wong, Andrew TL Tsang, Joe CT Koo, Leo LH Luk, Hui-Ling Yen, Malik Peris, Leo LM Poon

**Affiliations:** 1 School of Public Health, The University of Hong Kong, Hong Kong Special Administrative Region (Hong Kong SAR), China; 2 Government of the Hong Kong Special Administrative Region (HKSARG), Hong Kong SAR, China; 3 Asia Pacific Veterinary Information Services, Melbourne, Victoria, Australia; 4 Centre for Immunology and Infection, Hong Kong SAR, China; 5 HKU-Pasteur Research Pole, The University of Hong Kong, Hong Kong SAR, China; 6 HKJC Global Health Institute, The University of Hong Kong, Hong Kong SAR, China

**Keywords:** Avian influenza virus, H5N1, H5N8, surveillance and risk assessment

## Abstract

We isolated three genotypes of highly pathogenic avian influenza virus (HPAIV) clade 2.3.4.4b from wild birds infected with H5N1 (n = 12) and H5N8 (n = 1) in Hong Kong SAR 2021–2024. Viruses from two spoonbills from late 2022 were genetically related to a virus from a human in China. Four tested viruses exhibited variable virulence in mice but were susceptible to approved antivirals. No neutralising antibody was detected in 63 age-stratified human sera, suggesting potential risk should the virus adapt to humans.

First detected in China in 1996, highly pathogenic avian influenza viruses (HPAIV) A(H5N1) within the Goose/Guangdong1/96-lineage have genetically evolved, diverging into multiple clades. Viruses within clade 2.3.4.4b travelled via wild birds from Asia to Europe and Africa between 2016 and 2017. They returned to China in 2020 (H5N8) and 2021 (H5N1) persisting in wild bird populations, facilitating spread across all regions apart from Oceania [1]. Worryingly, H5N1 infections have been reported in mammals and humans across multiple continents. As such, these viruses pose a threat, considering the One Health perspective.

## Detection of H5 viruses in 13 wild bird samples

To aid in the risk assessment of clade 2.3.4.4b H5 viruses, we genetically and experimentally characterised HPAIV clade 2.3.4.4b H5 viruses isolated from 13 wild birds in the Hong Kong Special Administrative Region (Hong Kong SAR) between 2021 and 2024 (Table 1). By utilising embryonated chicken eggs for incubation and virus isolation [2], we isolated 12 H5N1 and one H5N8 influenza viruses from samples collected from carcasses of wild birds submitted to the Department of Agriculture, Fisheries and Conservation (AFCD), the Government of the HKSAR (HKSARG) (n = 10) or collected during winter-spring monthly wild bird surveillance in Mai Po Nature Reserve, conducted by the University of Hong Kong (HKU) (n = 3). Remarkably, three samples were collected from dead black-faced spoonbills (*Platalea minor*), a globally endangered species. We sequenced all these 13 H5-positive samples and submitted their genomic sequences to the Global Initiative on Sharing All Influenza Data (GISAID) database (https://gisaid.org/) (Table 1).

**Table 1:** Highly pathogenic avian influenza viruses isolated from wild birds, Hong Kong, 2021–2024 (n = 13)

Isolate	Abbreviation	Sampling date	Source	Bird species or sample type	Subtype	Cleavage sites	Genotype^a^	GISAID
A/Peregrine Falcon/Hong Kong/AFCD-HKU-21–013–27/2021	Peregrine_Falcon/21–01327	29 Jan 2021	AFCD	Peregrine falcon	H5N8	KRRKR	NA	EPI_ISL_19258149
A/Black Faced Spoonbill/Hong Kong/AFCD-HKU-21–17198.01012/2021	Spoonbill/21–17198	17 Dec 2021	AFCD	Black-faced spoonbill	H5N1	RRRKR	G7	EPI_ISL_19287787
A/Curlew/Hong Kong/AFCD-HKU-22–01095.01009/2022	Curlew/22–01095	26 Jan 2022	AFCD	Curlew	H5N1	RRRKR	G7	EPI_ISL_19258146
A/Black Faced Spoonbill/Hong Kong/AFCD-HKU-22–21429–01012/2022	Spoonbill/22–21429	24 Nov 2022	AFCD	Black-faced spoonbill	H5N1	KRRKR	G10	EPI_ISL_19258148
A/Environment/Hong Kong/HKUSPH_MP22_189A/2022	MP22_189A	9 Nov 2022	HKU	Environment	H5N1	RRRKR	G7	EPI_ISL_19258147
A/Black Faced Spoonbill/Hong Kong/AFCD-HKU-22–21944–01009/2022	Spoonbill/22–21944	6 Dec 2022	AFCD	Black-faced spoonbill	H5N1	KRRKR	G10	EPI_ISL_19135474
A/Environment/Hong Kong/HKUSPH_MP23_532P1/2023	MP23_532P1	6 Dec 2023	HKU	Environment	H5N1	KRRKR	G1	EPI_ISL_19135515
A/Eurasian Teal/Hong Kong/AFCD-HKU-23–14009–01020/2023	Eurasian_Teal/23–14009	11 Dec 2023	AFCD	Eurasian teal	H5N1	KRRKR	G1	EPI_ISL_19258154
A/Eurasian Teal/Hong Kong/AFCD-HKU-23–14383–02001/2023	Eurasian_Teal/23–14383	20 Dec 2023	AFCD	Eurasian teal	H5N1	KRRKR	G1	EPI_ISL_19258151
A/Gallinago Stenura/Hong Kong/AFCD-HKU-23–14383–01014/2023	Gallinago_Stenura/23–14383	20 Dec 2023	AFCD	Pin-tailed snipe	H5N1	KRRKR	G1	EPI_ISL_19258153
A/Eurasian Wigeon/Hong Kong/AFCD-HKU-23–14524–01009/2023	Eurasian_Wigeon/23–14524	22 Dec 2023	AFCD	Eurasian wigeon	H5N1	KRRKR	G1	EPI_ISL_19258150
A/Eurasian Wigeon/Hong Kong/AFCD-HKU-23–14484–01001/2023	Eurasian_Wigeon/23–14484	22 Dec 2023	AFCD	Eurasian wigeon	H5N1	KRRKR	G1	EPI_ISL_19258152
A/Environment/Hong Kong/HKUSPH_MP24_134P1b/2024	MP24_134P1b	6 Nov 2024	HKU	Environment	H5N1	KRRKR	G1	EPI_ISL_19640278

AFCD: Department of Agriculture, Fisheries and Conservation, Hong Kong; GISAID: Global Initiative on Sharing All Influenza Data; H: hemagglutinin; HKU: The University of Hong Kong Hong; N: neuraminidase; NA: not applicable.

^a^ Genotype based on [3]. These viruses were also classified using GenoFLU (https://GitHub.com/USDA-VS/GenoFLU). MP23_532P1, Eurasian_Teal/23–14009, Eurasian_Teal/23–14383, Gallinago_Stenura/23–14383, Eurasian_Wigeon/23–14524, Eurasian_Wigeon/23–14484, and MP24_134P1b are categorised as A3. MP22_189A is classified as A4, while the remaining are not assigned.

We performed phylogenetic analysis of all eight segments of these H5 isolates. Phylogenetic analysis revealed that these 13 HPAIVs formed four distinct subgroups within clade 2.3.4.4b, i.e. subgroups A, B, C and D (Figure) and Supplementary Figure with a different subgroup detected in each autumn/winter period. These isolates were genetically distinct from the H5 strains found in dairy cows in the United States (US) (Figure). The internal segment phylogeny trees can be seen in Supplementary Figure.

**Figure:**
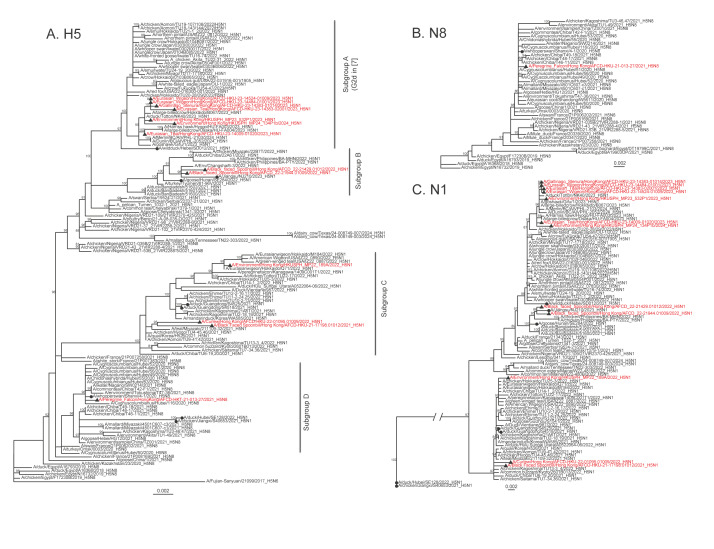
Phylogenetic trees of H5, N8 and N1 sequences from highly pathogenic avian influenza viruses isolated from Asia, North America, Europe and Africa, 2017–2024 (n = 261)

The maximum-likelihood phylogenetic trees were constructed by incorporating sequences from studied Hong Kong H5 viruses (represented by triangles and highlighted in red) along with related sequences from GISAID (https://gisaid.org/) and NCBI (https://www.ncbi.nlm.nih.gov/), as well as reference strains (represented by dots) [3]. The trees were built using IQTree with the GTR + G model and 1,000 bootstrap replicates. Bootstrap values exceeding 80 are displayed on the tree. Scale bars indicate estimated genetic distances.

The H5N1 wild bird isolates collected in 2023 and 2024 (subgroup A) were classified as genotype G1, based on the classification system used by Cui et al. [3], with the hemagglutinin (HA) gene clustering with Japanese samples in the G2d subgroup, which comprises HPAIVs isolated in Hokkaido during three consecutive seasons (2021–2023) and shares common ancestors with isolates from eastern Russia in spring and autumn 2022 [4]. Some US samples also fell into this cluster, indicating its presence across continents.

Three subgroup C isolates (MP22_189A, Spoonbill/21–17198 and Curlew/22–01095) exhibited high similarities to the H5 virus collected in a southern Japan outbreak, categorised in genotype G7, detected in different Asian countries between October 2021 and February 2022 [3]. Furthermore, our findings revealed the presence of US samples within genotype G7, indicating the existence of G7 in North America in 2022.

All the eight segments of our H5N8 virus (subgroup D) cluster with A/whooper swan/Shanxi/4–1/2020(H5N8) and viruses obtained from birds in Hubei, China – spreading from Siberia to central China with migratory birds along the East Asian-Australasian flyway [5].

The remaining two isolates (subgroup B), collected from black-faced spoonbill in 2022, exhibited high nt identities with a human isolate A/Jiangsu/NJ210/2023 (ranging from 98.9% to 99.6%) from China and were classified as G10 (Table 1). The genome similarity information between these sequences can be found in Supplementary Table 1. This genotype originated from Russia and was detected in Russia, China and South Korea [4]. The L325P mutation in the HA protein represents a genomic disparity between A/Jiangsu/NJ210/2023 and Spoonbill/22–21944. This mutation, previously observed in a mouse-adapted H5N8 virus, underscores the necessity for further in-depth analysis [6].

Mutations related to an increased affinity for human α2–6 glycans were identified in the 13 studied H5 viruses (e.g. T160A, D187N, K193N/D and Q196K (H3 numbering) in the HA gene) [7,8]. These mutations locate in the antigenic regions of H5 HA (Sites A and B) [9], but the impact of these mutations on the antigenicity of the virus still needs to be experimentally determined. Genotype G10 isolates have mutations that may increase the mammalian adaptation (HA-N158D, PB2-I292V, PA-V63I and PA-Q400P) and the pathogenesis (NP-M105V, PB1-F2-N66S) [10-13]. The mammalian adaptation or pathogenesis-related mutations of these H5 viruses are detailed in Supplementary Table 2.

## Experimental characterisation of H5N1 viruses detected in Hong Kong

Considering the high genetic similarity between our G10 and human H5N1(A/Jiangsu/NJ210/2023) viruses, we experimentally characterised two 2022 isolates, including the G10 virus (Spoonbill/22–21429) and G7 virus (Curlew/22–01095) and two 2023 isolates, including G1 viruses (Eurasian Teal/23–14009 and MP23_532P1). In mice, the studied G1 viruses were more virulent than the studied G10 and G7 viruses, with 1,000-fold difference in the determined 50% mouse lethal dose (MLD_50_) (Table 2). Three of the four tested viruses were neurotropic, while the viral load detected in the lungs were comparable.

**Table 2:** Pathogenicity in mouse tissues and antiviral susceptibility in vitro of clade 2.3.4.4b highly pathogenic avian influenza virus H5N1 isolates, Hong Kong, 2022–2023 (n = 4)

Isolate	Genotype	Mice (n)	Virus titres in mouse tissues (log_10_TCID_50_/mL)										log_10_MLD_50_/mL	1 MLD_50_ in TCID_50_	Oseltamivir (nM)			Zanamivir (nM)			Baloxavir acid (nM)	
			Lungs, 3 dpi			Lungs, 5 dpi			Brains, 3 dpi	Brains, 5 dpi					IC_50_	SD	Fold change	IC_50_	SD	Fold change	EC_50_	SD
			Positive (n)	Mean	SD	Positive (n)	Mean	SD	Mean	Positive (n)	Mean	SD										
Curlew/22–01095	G7	3	3	4.3	0.5	3	6.2	0.7	< 1.79	1	2.9	1.9	3.9	20,629.1	13.0	1.3	9.4	0.6	0.1	0.7	1.7	0.3
Spoonbill/22–21429	G10	3	3	3.5	0.3	3	5.2	1.0	< 1.79	0	< 1.79		3.9	53,886.5	9.2	2.2	6.7	0.9	0.0	1.0	1.3	0.4
Eurasian_Teal/23–14009	G1	4	4	3.9	0.3	4	5.3	0.4	< 1.79	4	6.0	0.9	6.9	69.7	19.7	2.6	14.2	0.8	0.1	1.0	1.3	0.7
MP23_532P1	G1	4	4	3.9	0.2	4	5.0	0.3	< 1.79	2	3.2	1.6	6.8	329.8	15.7	2.4	11.3	0.9	0.1	1.0	1.7	0.6

dpi: days post infection; EC_50_: half-maximal effective concentration; IC_50_: half-maximal inhibitory concentration; MLD_50_: 50% mouse lethal dose; SD: standard deviation; TCID_50_: 50% tissue culture infectious dose.

We further examined the susceptibility of these four viruses to approved antivirals (Table 2).

The four viruses exhibited comparable susceptibility to neuraminidase (NA) inhibitors (oseltamivir and zanamivir) and polymerase acidic (PA) endonuclease inhibitor (baloxavir acid). We conducted hemagglutination inhibition and neutralising antibody tests against G10, utilising age-stratified human sera. The sample set comprised 63 specimens collected by the Hong Kong Red Cross. They were collected from healthy blood donors, aged 16–79 years, between April and December 2020. The samples were grouped by decade for testing, in accordance with World Health Organization (WHO) guidelines for serology tests. None of the tested human samples were found to be positive in these assays. The detailed information on the seroprevalence is presented in Supplementary Table 3.

## Discussion

Avian influenza A(H5N1) viruses within clade 2.3.4.4b have infected multiple mammalian species (e.g. sea mammals, farmed mink, cats and cattle) in different continents in recent years [14,15]. Moreover, clade 2.3.4.4b H5 viruses have caused human infection in several countries, including Chile, the US, the United Kingdom (UK), Russia, China and Vietnam [16]. We identified 13 HPAIVs from avian samples in Hong Kong between January 2021 and November 2024. The phylogenetic analysis revealed that the H5N8 virus isolated in 2021 clustered with samples from wild birds in Hubei Province, China. Additionally, we observed the presence of G7 and G1 strains in Hong Kong, showing high genetic similarities to H5 strains from Korea and Japan, with some US samples in 2022 also falling into these two virus groups. That means these viruses can spread in different continents. Notably, the HPAIV isolated in 2023 and 2024 belonged exclusively to the G2d group, which has been repeatedly detected in Japan for three consecutive years. These findings underscore Hong Kong's critical role in HPAI H5 virus transmission within the East Asian-Australasian flyway and emphasise the importance of it being a strategic site for avian influenza surveillance. Genotype G1 H5N1 viruses were also detected in multiple countries across Europe, Africa, Asia and North America [3]. Further surveillance in wild birds in the East Asian-Australasian and other flyways might help to understand the transmission dynamic of H5 viruses between different geographical locations.

Of note, we detected G10 strains in Hong Kong, following their initial identification in Russia in October 2021 and subsequent spread to central China by December 2021. The G10 strains were close to a human influenza virus isolate and exhibited high pathogenicity in mice, with amino acid residues potentially enhancing their binding to human receptors and increasing virulence in mammals. Importantly, we noticed that the G1 virus, which is present in in Europe, Africa, Asia and North America, demonstrated more virulence than the G10 virus in mice. Interestingly, G1 and G10 viruses have PB2 segments from different origins [3]. Further characterisation of PB2 or other gene segments among these viruses is warranted.

We did not find neutralising antibodies to the virus in human sera, suggesting a potential outbreak risk should the virus adapt to humans.

## Conclusion

We isolated 13 clade 2.3.4.4b H5 viruses in Hong Kong, and these viruses were genetically distinct from the H5 strains found in the outbreak in dairy cattle in the US. Given the pathogenicity and potential transmission of these avian viruses in mammals, it is crucial to implement the One Health strategy. Our study emphasises the necessity of continued surveillance and control measures to prevent potential outbreaks and transmission to both humans and other animals.

## Supplementary Data

510000 Supplementary Material
