# Supplementary material for: Detection and characterisation of high pathogenicity avian influenza virus (H5N1/H5N8) clade 2.3.4.4b, Hong Kong SAR, China, 2021 to 2024
Source: Euro Surveill. 2025 Jan 9;30(1):2400839. doi: 10.2807/1560-7917.ES.2025.30.1.2400839 (PMC11719804; doi:10.2807/1560-7917.ES.2025.30.1.2400839)
Supplement: Supplementary Material [file 24-00839_POON_Supplement.pdf]

Detection and characterisation of high pathogenicity avian influenza virus (H5N1/H5N8) clade 2.3.4.4b, Hong Kong, 2021 to 2024

This supplementary material is hosted by Eurosurveillance as supporting information alongside the article [Detection and characterisation of high pathogenicity avian influenza virus (H5N1/H5N8) clade 2.3.4.4b, Hong Kong, 2021 to 2024], on behalf of the authors, who remain responsible for the accuracy and appropriateness of the content. The same standards for ethics, copyright, attributions and permissions as for the article apply. Supplements are not edited by Eurosurveillance and the journal is not responsible for the maintenance of any links or email addresses provided therein.

Supplementary Figure

Supplementary Figure A-F. The maximum-likelihood phylogenetic tree of H5Nx viruses includes sequences from Hong Kong H5 viruses (depicted in red), related sequences from GISAID and NCBI databases, and reference strains (depicted in blue) (S1:PB2, S2:PB1, S3:PA, S4:NP, S5:M and S6:NS. The tree was generated using IQtree with the GTR+G model and 1000 bootstrap replicates. Bootstrap values greater than 80 are indicated on the tree. Scale bars indicate estimated genetic distances.

A) PB2

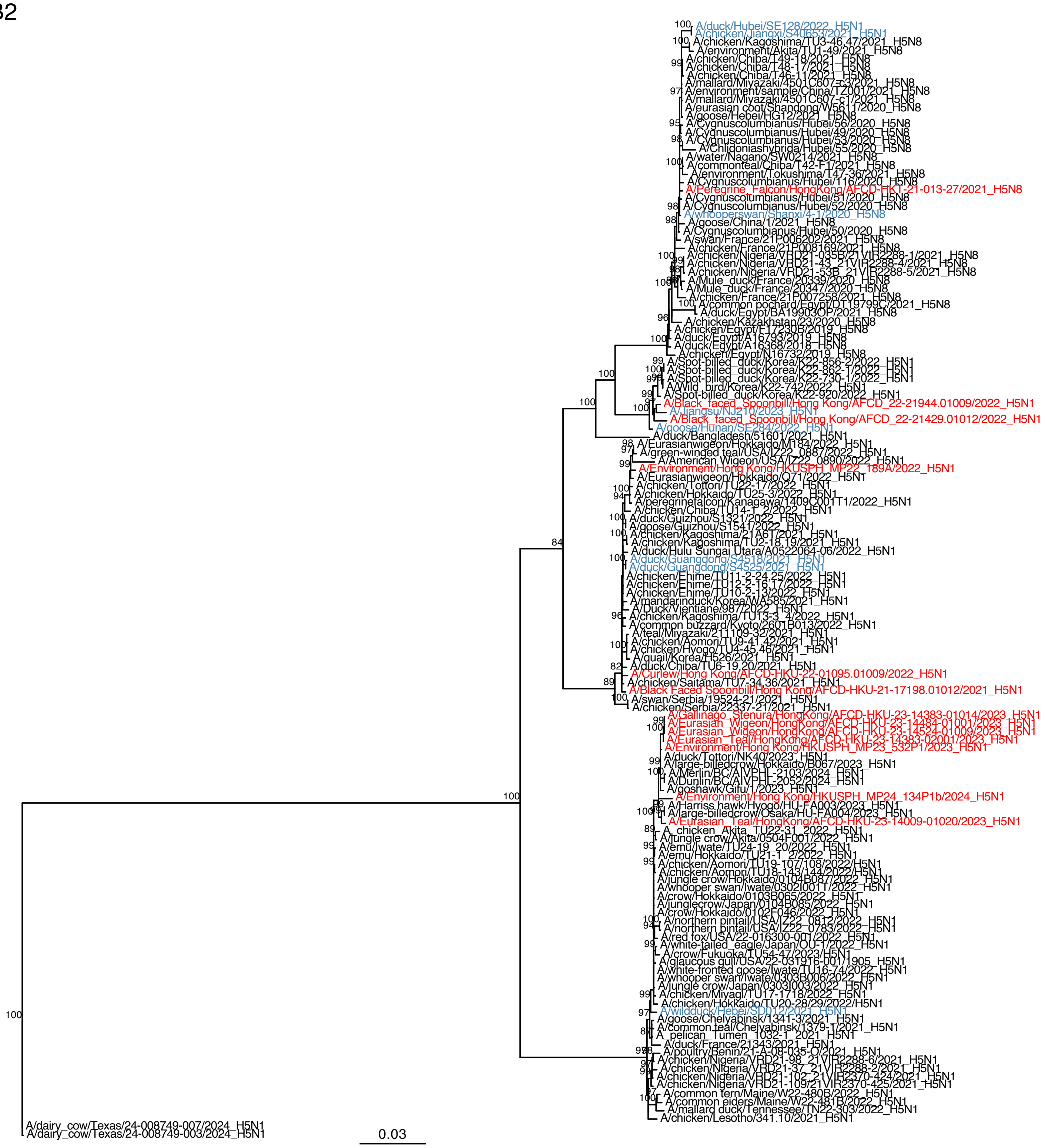

A/duck/Hubei/SE128/2022_H5N1
A/chicken/Jiangxi/S40653/2021_H5N1
A/chicken/Kagoshima/TU3-46,47/2021_H5N8
A/environment/Akita/TU1-49/2021_H5N8
A/chicken/Chiba/T49-18/2021_H5N8
A/chicken/Chiba/T48-17/2021_H5N8
A/chicken/Chiba/T46-11/2021_H5N8
A/mallard/Miyazaki/4501C607-c3/2021_H5N8
A/environment/sample/China/TZ001/2021_H5N8
A/mallard/Miyazaki/4501C607-c1/2021_H5N8
A/eurasian coot/Shandong/W5611/2020_H5N8
A/goose/Hebei/HG12/2021_H5N8
A/Cygnuscolumbianus/Hubei/56/2020_H5N8
A/Cygnuscolumbianus/Hubei/49/2020_H5N8
A/Cygnuscolumbianus/Hubei/53/2020_H5N8
A/Chlidoniashybrida/Hubei/55/2020_H5N8
A/water/Nagano/SW0214/2021_H5N8
A/commonteal/Chiba/T42-F1/2021_H5N8
A/environment/Tokushima/T47-36/2021_H5N8
A/Cygnuscolumbianus/Hubei/116/2020_H5N8
A/Peregrine_Falcon/HongKong/AFCD-HKT-21-013-27/2021_H5N8
A/Cygnuscolumbianus/Hubei/51/2020_H5N8
A/Cygnuscolumbianus/Hubei/52/2020_H5N8
A/whooperswan/Shanxi/4-1/2020_H5N8
A/goose/China/1/2021_H5N8
A/Cygnuscolumbianus/Hubei/50/2020_H5N8
A/swan/France/21P006202/2021_H5N8
A/chicken/France/21P008169/2021_H5N8
A/chicken/Nigeria/VRD21-035B/21VIR2288-1/2021_H5N8
A/chicken/Nigeria/VRD21-43_21VIR2288-4/2021_H5N8
A/chicken/Nigeria/VRD21-53B_21VIR2288-5/2021_H5N8
A/Mule_duck/France/20339/2020_H5N8
A/Mule_duck/France/20347/2020_H5N8
A/chicken/France/21P007258/2021_H5N8
A/common pochard/Egypt/DT19799C/2021_H5N8
A/duck/Egypt/BA19903OP/2021_H5N8
A/chicken/Kazakhstan/23/2020_H5N8
A/chicken/Egypt/F17230B/2019_H5N8
A/duck/Egypt/A16793/2019_H5N8
A/duck/Egypt/A16368/2018_H5N8
A/chicken/Egypt/N16732/2019_H5N8
A/Spot-billed_duck/Korea/K22-856-2/2022_H5N1
A/Spot-billed_duck/Korea/K22-862-1/2022_H5N1
A/Spot-billed_duck/Korea/K22-730-1/2022_H5N1
A/Wild_bird/Korea/K22-742/2022_H5N1
A/Spot-billed_duck/Korea/K22-920/2022_H5N1
A/Black_faced_Spoonbill/Hong Kong/AFCD_22-21944.01009/2022_H5N1
A/Jiangsu/NJ210/2023_H5N1
A/Black_faced_Spoonbill/Hong Kong/AFCD_22-21429.01012/2022_H5N1
A/goose/Hunan/SE284/2022_H5N1
A/duck/Bangladesh/51601/2021_H5N1
A/Eurasianwigeon/Hokkaido/M184/2022_H5N1
A/green-winged teal/USA/IZ22_0887/2022_H5N1
A/American Wigeon/USA/IZ22_0890/2022_H5N1
A/Environment/Hong Kong/HKUSPH_MP22_189A/2022_H5N1
A/Eurasianwigeon/Hokkaido/Q71/2022_H5N1
A/chicken/Tottori/TU22-17/2022_H5N1
A/chicken/Hokkaido/TU25-3/2022_H5N1
A/peregrinefalcon/Kanagawa/1409C001T1/2022_H5N1
A/chicken/Chiba/TU14-1_2/2022_H5N1
A/duck/Guizhou/S1321/2022_H5N1
A/goose/Guizhou/S1541/2022_H5N1
A/chicken/Kagoshima/21A6T/2021_H5N1
A/chicken/Kagoshima/TU2-18,19/2021_H5N1
A/duck/Hulu Sungai Utara/A0522064-06/2022_H5N1
A/duck/Guangdong/S4518/2021_H5N1
A/duck/Guangdong/S4525/2021_H5N1
A/chicken/Ehime/TU11-2-24,25/2022_H5N1
A/chicken/Ehime/TU12-2-16,17/2022_H5N1
A/chicken/Ehime/TU10-2-13/2022_H5N1
A/mandarinduck/Korea/WA585/2021_H5N1
A/Duck/Vientiane/987/2022_H5N1
A/chicken/Kagoshima/TU13-3_4/2022_H5N1
A/common buzzard/Kyoto/2601B013/2022_H5N1
A/teal/Miyazaki/211109-32/2021_H5N1
A/chicken/Aomori/TU9-41,42/2021_H5N1
A/chicken/Hyogo/TU4-45,46/2021_H5N1
A/quail/Korea/H526/2021_H5N1
A/duck/Chiba/TU6-19,20/2021_H5N1
A/Curlew/Hong Kong/AFCD-HKU-22-01095.01009/2022_H5N1
A/chicken/Saitama/TU7-34,36/2021_H5N1
A/Black Faced Spoonbill/Hong Kong/AFCD-HKU-21-17198.01012/2021_H5N1
A/swan/Serbia/19524-21/2021_H5N1
A/chicken/Serbia/22337-21/2021_H5N1
A/Gallinago_Stenura/HongKong/AFCD-HKU-23-14383-01014/2023_H5N1
A/Eurasian_Wigeon/HongKong/AFCD-HKU-23-14484-01001/2023_H5N1
A/Eurasian_Wigeon/HongKong/AFCD-HKU-23-14524-01009/2023_H5N1
A/Eurasian_Teal/HongKong/AFCD-HKU-23-14383-02001/2023_H5N1
A/Environment/Hong Kong/HKUSPH_MP23_532P1/2023_H5N1
A/duck/Tottori/NK40/2023_H5N1
A/large-billedcrow/Hokkaido/B067/2023_H5N1
A/Merlin/BC/AIVPHL-2103/2024_H5N1
A/Dunlin/BC/AIVPHL-2052/2024_H5N1
A/goshawk/Gifu/1/2023_H5N1
A/Environment/Hong Kong/HKUSPH_MP24_134P1b/2024_H5N1
A/Harriss hawk/Hyogo/HU-FA003/2023_H5N1
A/large-billedcrow/Osaka/HU-FA004/2023_H5N1
A/Eurasian_Teal/HongKong/AFCD-HKU-23-14009-01020/2023_H5N1
A_chicken_Akita_TU22-31_2022_H5N1
A/jungle crow/Akita/0504F001/2022_H5N1
A/emu/Iwate/TU24-19_20/2022_H5N1
A/emu/Hokkaido/TU21-1_2/2022_H5N1
A/chicken/Aomori/TU19-107/108/2022/H5N1
A/chicken/Aomori/TU18-143/144/2022/H5N1
A/jungle crow/Hokkaido/0104B087/2022_H5N1
A/whooper swan/Iwate/0302I001T/2022_H5N1
A/crow/Hokkaido/0103B065/2022_H5N1
A/junglecrow/Japan/0104B085/2022_H5N1
A/crow/Hokkaido/0102F046/2022_H5N1
A/northern pintail/USA/IZ22_0812/2022_H5N1
A/northern pintail/USA/IZ22_0783/2022_H5N1
A/red fox/USA/22-016300-001/2022_H5N1
A/white-tailed_eagle/Japan/OU-1/2022_H5N1
A/crow/Fukuoka/TU54-47/2023/H5N1
A/glaucous gull/USA/22-031916-001/1905_H5N1
A/white-fronted goose/Iwate/TU16-74/2022_H5N1
A/whooper swan/Iwate/0303B006/2022_H5N1
A/jungle crow/Japan/0303I003/2022_H5N1
A/chicken/Miyagi/TU17-1718/2022_H5N1
A/chicken/Hokkaido/TU20-28/29/2022/H5N1
A/wildduck/Hebei/SD012/2021_H5N1
A/goose/Chelyabinsk/1341-3/2021_H5N1
A/common teal/Chelyabinsk/1379-1/2021_H5N1
A_pelican_Tumen_1032-1_2021_H5N1
A/duck/France/21343/2021_H5N1
A/poultry/Benin/21-A-08-035-O/2021_H5N1
A/chicken/Nigeria/VRD21-98_21VIR2288-6/2021_H5N1
A/chicken/Nigeria/VRD21-37_21VIR2288-2/2021_H5N1
A/chicken/Nigeria/VRD21-102_21VIR2370-424/2021_H5N1
A/chicken/Nigeria/VRD21-109/21VIR2370-425/2021_H5N1
A/common tern/Maine/W22-480B/2022_H5N1
A/common eiders/Maine/W22-481B/2022_H5N1
A/mallard duck/Tennessee/TN22-303/2022_H5N1
A/chicken/Lesotho/341.10/2021_H5N1
A/dairy_cow/Texas/24-008749-007/2024_H5N1
A/dairy_cow/Texas/24-008749-003/2024_H5N1
0.03

B) PB1

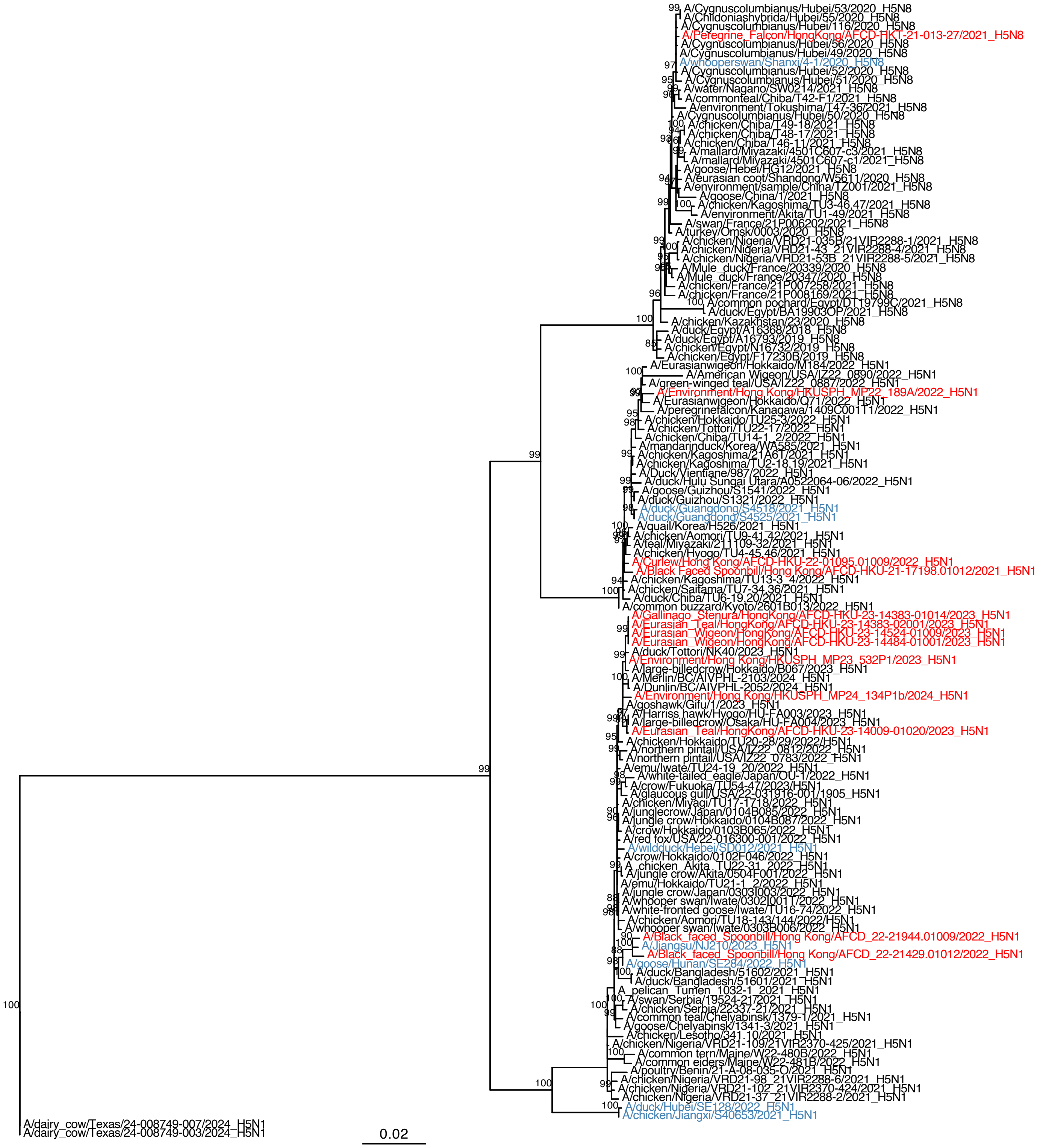

C) PA

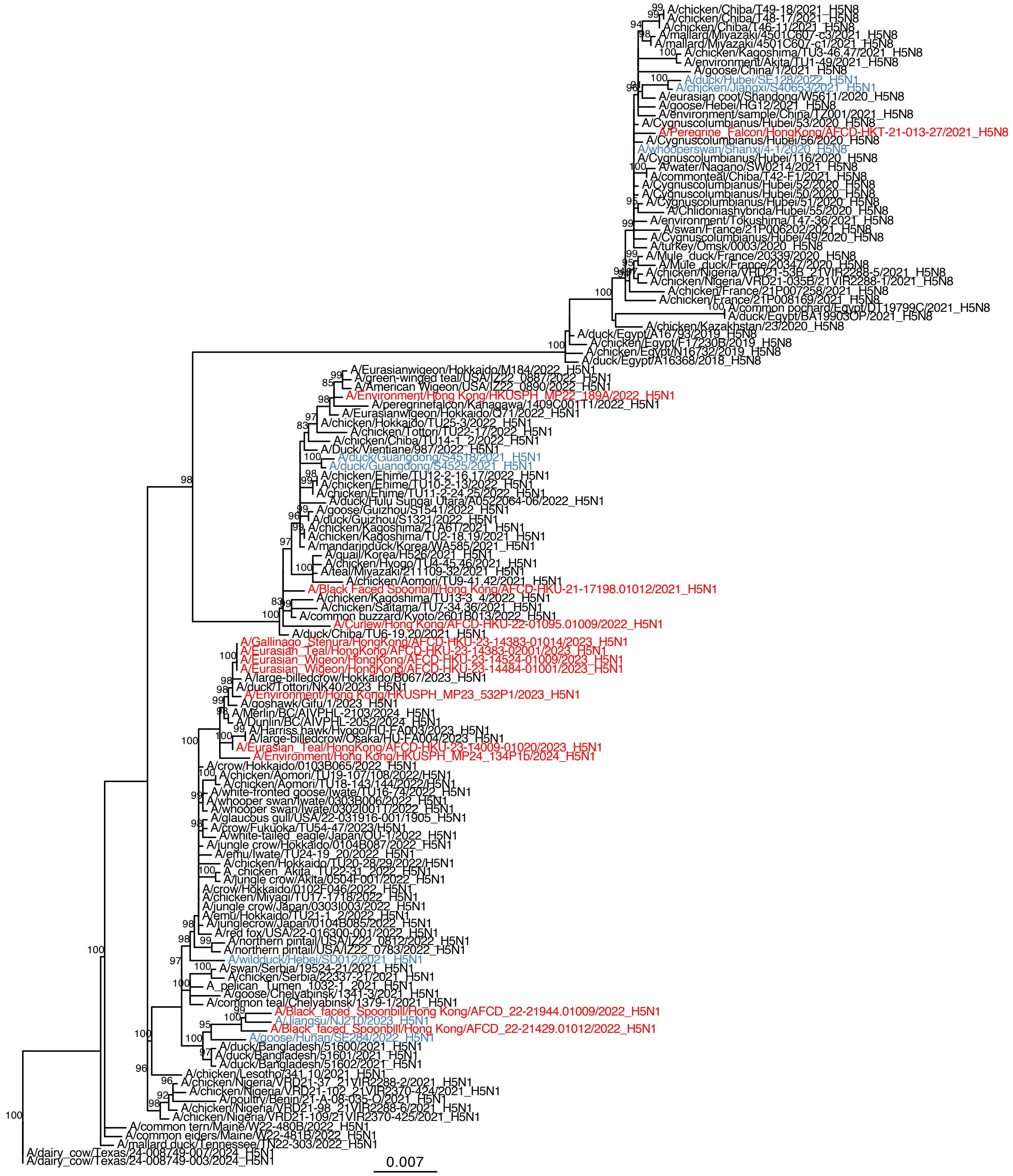
A/chicken/Chiba/T49-18/2021_H5N8
A/chicken/Chiba/T48-17/2021_H5N8
A/chicken/Chiba/T46-11/2021_H5N8
A/mallard/Miyazaki/4501C607-c3/2021_H5N8
A/mallard/Miyazaki/4501C607-c1/2021_H5N8
A/chicken/Kagoshima/TU3-46,47/2021_H5N8
A/environment/Akita/TU1-49/2021_H5N8
A/goose/China/1/2021_H5N8
A/duck/Hubei/SE128/2022_H5N1
A/chicken/Jiangxi/S40653/2021_H5N1
A/eurasian coot/Shandong/W5611/2020_H5N8
A/goose/Hebei/HG12/2021_H5N8
A/environment/sample/China/TZ001/2021_H5N8
A/Cygnuscolumbianus/Hubei/53/2020_H5N8
A/Peregrine_Falcon/HongKong/AFCD-HKT-21-013-27/2021_H5N8
A/Cygnuscolumbianus/Hubei/56/2020_H5N8
A/whooperswan/Shanxi/4-1/2020_H5N8
A/Cygnuscolumbianus/Hubei/116/2020_H5N8
A/water/Nagano/SW0214/2021_H5N8
A/commonteal/Chiba/T42-F1/2021_H5N8
A/Cygnuscolumbianus/Hubei/52/2020_H5N8
A/Cygnuscolumbianus/Hubei/50/2020_H5N8
A/Cygnuscolumbianus/Hubei/51/2020_H5N8
A/Chlidoniashybrida/Hubei/55/2020_H5N8
A/environment/Tokushima/T47-36/2021_H5N8
A/swan/France/21P006202/2021_H5N8
A/Cygnuscolumbianus/Hubei/49/2020_H5N8
A/turkey/Omsk/0003/2020_H5N8
A/Mule_duck/France/20339/2020_H5N8
A/Mule_duck/France/20347/2020_H5N8
A/chicken/Nigeria/VRD21-53B_21VIR2288-5/2021_H5N8
A/chicken/Nigeria/VRD21-035B/21VIR2288-1/2021_H5N8
A/chicken/France/21P007258/2021_H5N8
A/chicken/France/21P008169/2021_H5N8
A/common pochard/Egypt/DT19799C/2021_H5N8
A/duck/Egypt/BA19903OP/2021_H5N8
A/chicken/Kazakhstan/23/2020_H5N8
A/duck/Egypt/A16793/2019_H5N8
A/chicken/Egypt/F17230B/2019_H5N8
A/chicken/Egypt/N16732/2019_H5N8
A/duck/Egypt/A16368/2018_H5N8
A/Eurasianwigeon/Hokkaido/M184/2022_H5N1
A/green-winged teal/USA/IZ22_0887/2022_H5N1
A/American Wigeon/USA/IZ22_0890/2022_H5N1
A/Environment/Hong Kong/HKUSPH_MP22_189A/2022_H5N1
A/peregrinefalcon/Kanagawa/1409C001T1/2022_H5N1
A/Eurasianwigeon/Hokkaido/Q71/2022_H5N1
A/chicken/Hokkaido/TU25-3/2022_H5N1
A/chicken/Tottori/TU22-17/2022_H5N1
A/chicken/Chiba/TU14-1_2/2022_H5N1
A/Duck/Vientiane/987/2022_H5N1
A/duck/Guangdong/S4518/2021_H5N1
A/duck/Guangdong/S4525/2021_H5N1
A/chicken/Ehime/TU12-2-16,17/2022_H5N1
A/chicken/Ehime/TU10-2-13/2022_H5N1
A/chicken/Ehime/TU11-2-24,25/2022_H5N1
A/duck/Hulu Sungai Utara/A0522064-06/2022_H5N1
A/goose/Guizhou/S1541/2022_H5N1
A/duck/Guizhou/S1321/2022_H5N1
A/chicken/Kagoshima/21A6T/2021_H5N1
A/chicken/Kagoshima/TU2-18,19/2021_H5N1
A/mandarinduck/Korea/WA585/2021_H5N1
A/quail/Korea/H526/2021_H5N1
A/chicken/Hyogo/TU4-45,46/2021_H5N1
A/teal/Miyazaki/211109-32/2021_H5N1
A/chicken/Aomori/TU9-41,42/2021_H5N1
A/Black Faced Spoonbill/Hong Kong/AFCD-HKU-21-17198.01012/2021_H5N1
A/chicken/Kagoshima/TU13-3_4/2022_H5N1
A/chicken/Saitama/TU7-34,36/2021_H5N1
A/common buzzard/Kyoto/2601B013/2022_H5N1
A/Curlew/Hong Kong/AFCD-HKU-22-01095.01009/2022_H5N1
A/duck/Chiba/TU6-19,20/2021_H5N1
A/Gallinago_Stenura/HongKong/AFCD-HKU-23-14383-01014/2023_H5N1
A/Eurasian_Teal/HongKong/AFCD-HKU-23-14383-02001/2023_H5N1
A/Eurasian_Wigeon/HongKong/AFCD-HKU-23-14524-01009/2023_H5N1
A/Eurasian_Wigeon/HongKong/AFCD-HKU-23-14484-01001/2023_H5N1
A/large-billedcrow/Hokkaido/B067/2023_H5N1
A/duck/Tottori/NK40/2023_H5N1
A/Environment/Hong Kong/HKUSPH_MP23_532P1/2023_H5N1
A/goshawk/Gifu/1/2023_H5N1
A/Merlin/BC/AIVPHL-2103/2024_H5N1
A/Dunlin/BC/AIVPHL-2052/2024_H5N1
A/Harriss hawk/Hyogo/HU-FA003/2023_H5N1
A/large-billedcrow/Osaka/HU-FA004/2023_H5N1
A/Eurasian_Teal/HongKong/AFCD-HKU-23-14009-01020/2023_H5N1
A/Environment/Hong Kong/HKUSPH_MP24_134P1b/2024_H5N1
A/crow/Hokkaido/0103B065/2022_H5N1
A/chicken/Aomori/TU19-107/108/2022/H5N1
A/chicken/Aomori/TU18-143/144/2022/H5N1
A/white-fronted goose/Iwate/TU16-74/2022_H5N1
A/whooper swan/Iwate/0303B006/2022_H5N1
A/whooper swan/Iwate/0302I001T/2022_H5N1
A/glaucous gull/USA/22-031916-001/1905_H5N1
A/crow/Fukuoka/TU54-47/2023/H5N1
A/white-tailed_eagle/Japan/OU-1/2022_H5N1
A/jungle crow/Hokkaido/0104B087/2022_H5N1
A/emu/Iwate/TU24-19_20/2022_H5N1
A/chicken/Hokkaido/TU20-28/29/2022/H5N1
A_chicken_Akita_TU22-31_2022_H5N1
A/jungle crow/Akita/0504F001/2022_H5N1
A/crow/Hokkaido/0102F046/2022_H5N1
A/chicken/Miyagi/TU17-17/18/2022_H5N1
A/jungle crow/Japan/0303I003/2022_H5N1
A/emu/Hokkaido/TU21-1_2/2022_H5N1
A/junglecrow/Japan/0104B085/2022_H5N1
A/red fox/USA/22-016300-001/2022_H5N1
A/northern pintail/USA/IZ22_0812/2022_H5N1
A/northern pintail/USA/IZ22_0783/2022_H5N1
A/wildduck/Hebei/SD012/2021_H5N1
A/swan/Serbia/19524-21/2021_H5N1
A/chicken/Serbia/22337-21/2021_H5N1
A_pelican_Tumen_1032-1_2021_H5N1
A/goose/Chelyabinsk/1341-3/2021_H5N1
A/common teal/Chelyabinsk/1379-1/2021_H5N1
A/Black_faced_Spoonbill/Hong Kong/AFCD_22-21944.01009/2022_H5N1
A/Jiangsu/NJ210/2023_H5N1
A/Black_faced_Spoonbill/Hong Kong/AFCD_22-21429.01012/2022_H5N1
A/goose/Hunan/SE284/2022_H5N1
A/duck/Bangladesh/51600/2021_H5N1
A/duck/Bangladesh/51601/2021_H5N1
A/duck/Bangladesh/51602/2021_H5N1
A/chicken/Lesotho/341.10/2021_H5N1
A/chicken/Nigeria/VRD21-37_21VIR2288-2/2021_H5N1
A/chicken/Nigeria/VRD21-102_21VIR2370-424/2021_H5N1
A/poultry/Benin/21-A-08-035-O/2021_H5N1
A/chicken/Nigeria/VRD21-98_21VIR2288-6/2021_H5N1
A/chicken/Nigeria/VRD21-109/21VIR2370-425/2021_H5N1
A/common tern/Maine/W22-480B/2022_H5N1
A/common eiders/Maine/W22-481B/2022_H5N1
A/mallard duck/Tennessee/TN22-303/2022_H5N1
A/dairy_cow/Texas/24-008749-007/2024_H5N1
A/dairy_cow/Texas/24-008749-003/2024_H5N1
0.007

D) NP

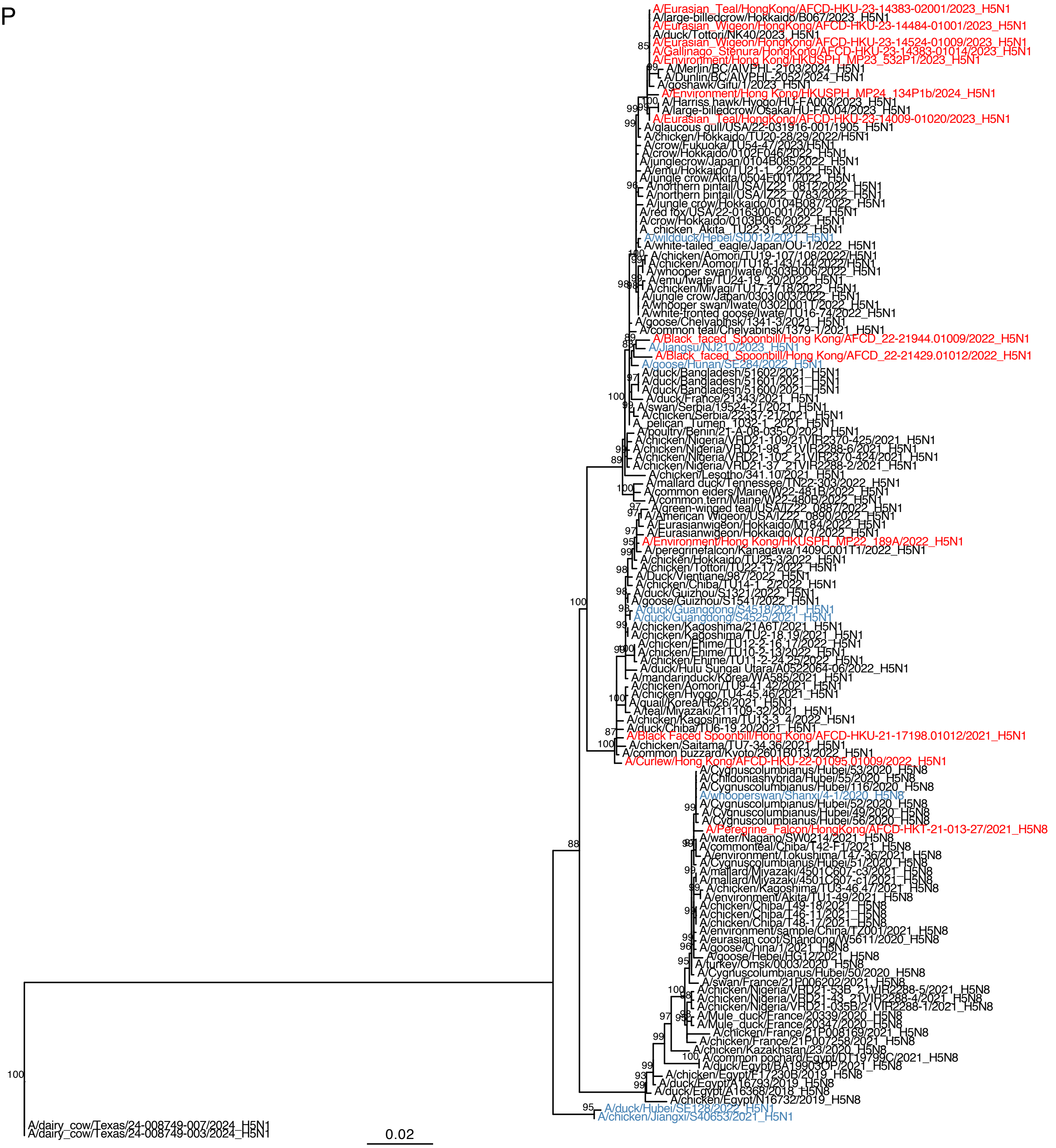

A/Eurasian_Teal/HongKong/AFCD-HKU-23-14383-02001/2023_H5N1
A/large-billedcrow/Hokkaido/B067/2023_H5N1
A/Eurasian_Wigeon/HongKong/AFCD-HKU-23-14484-01001/2023_H5N1
A/duck/Tottori/NK40/2023_H5N1
A/Eurasian_Wigeon/HongKong/AFCD-HKU-23-14524-01009/2023_H5N1
A/Gallinago_Stenura/HongKong/AFCD-HKU-23-14383-01014/2023_H5N1
A/Environment/Hong Kong/HKUSPH_MP23_532P1/2023_H5N1
A/Merlin/BC/AIVPHL-2103/2024_H5N1
A/Dunlin/BC/AIVPHL-2052/2024_H5N1
A/goshawk/Gifu/1/2023_H5N1
A/Environment/Hong Kong/HKUSPH_MP24_134P1b/2024_H5N1
A/Harriss hawk/Hyogo/HU-FA003/2023_H5N1
A/large-billedcrow/Osaka/HU-FA004/2023_H5N1
A/Eurasian_Teal/HongKong/AFCD-HKU-23-14009-01020/2023_H5N1
A/glaucous gull/USA/22-031916-001/1905_H5N1
A/chicken/Hokkaido/TU20-28/29/2022/H5N1
A/crow/Fukuoka/TU54-47/2023/H5N1
A/crow/Hokkaido/0102F046/2022_H5N1
A/junglecrow/Japan/0104B085/2022_H5N1
A/emu/Hokkaido/TU21-1_2/2022_H5N1
A/jungle crow/Akita/0504E001/2022_H5N1
A/northern pintail/USA/IZ22_0812/2022_H5N1
A/northern pintail/USA/IZ22_0783/2022_H5N1
A/jungle crow/Hokkaido/0104B087/2022_H5N1
A/red fox/USA/22-016300-001/2022_H5N1
A/crow/Hokkaido/0103B065/2022_H5N1
A_chicken_Akita_TU22-31_2022_H5N1
A/wildduck/Hebei/SD012/2021_H5N1
A/white-tailed_eagle/Japan/OU-1/2022_H5N1
A/chicken/Aomori/TU19-107/108/2022/H5N1
A/chicken/Aomori/TU18-143/144/2022/H5N1
A/whooper swan/Iwate/0303B006/2022_H5N1
A/emu/Iwate/TU24-19_20/2022_H5N1
A/chicken/Miyagi/TU17-17/18/2022_H5N1
A/jungle crow/Japan/0303I003/2022_H5N1
A/whooper swan/Iwate/0302I001T/2022_H5N1
A/white-fronted goose/Iwate/TU16-74/2022_H5N1
A/goose/Chelyabinsk/1341-3/2021_H5N1
A/common teal/Chelyabinsk/1379-1/2021_H5N1
A/Black_faced_Spoonbill/Hong Kong/AFCD_22-21944.01009/2022_H5N1
A/Jiangsu/NJ210/2023_H5N1
A/Black_faced_Spoonbill/Hong Kong/AFCD_22-21429.01012/2022_H5N1
A/goose/Hunan/SE284/2022_H5N1
A/duck/Bangladesh/51602/2021_H5N1
A/duck/Bangladesh/51601/2021_H5N1
A/duck/Bangladesh/51600/2021_H5N1
A/duck/France/21343/2021_H5N1
A/swan/Serbia/19524-21/2021_H5N1
A/chicken/Serbia/22237-21/2021_H5N1
A_pelican_Tumen_1032-1_2021_H5N1
A/poultry/Benin/21-A-08-035-O/2021_H5N1
A/chicken/Nigeria/VRD21-109/21VIR2370-425/2021_H5N1
A/chicken/Nigeria/VRD21-98_21VIR2288-6/2021_H5N1
A/chicken/Nigeria/VRD21-102_21VIR2370-424/2021_H5N1
A/chicken/Nigeria/VRD21-37_21VIR2288-2/2021_H5N1
A/chicken/Lesotho/341.10/2021_H5N1
A/mallard duck/Tennessee/TN22-303/2022_H5N1
A/common eiders/Maine/W22-481B/2022_H5N1
A/common tern/Maine/W22-480B/2022_H5N1
A/green-winged teal/USA/IZ22_0887/2022_H5N1
A/American Wigeon/USA/IZ22_0890/2022_H5N1
A/Eurasianwigeon/Hokkaido/M184/2022_H5N1
A/Eurasianwigeon/Hokkaido/Q71/2022_H5N1
A/Environment/Hong Kong/HKUSPH_MP22_189A/2022_H5N1
A/peregrinefalcon/Kanagawa/1409C001T1/2022_H5N1
A/chicken/Hokkaido/TU25-3/2022_H5N1
A/chicken/Tottori/TU22-17/2022_H5N1
A/Duck/Vientiane/987/2022_H5N1
A/chicken/Chiba/TU14-1_2/2022_H5N1
A/duck/Guizhou/S1321/2022_H5N1
A/goose/Guizhou/S1541/2022_H5N1
A/duck/Guangdong/S4518/2021_H5N1
A/duck/Guangdong/S4525/2021_H5N1
A/chicken/Kagoshima/21A6T/2021_H5N1
A/chicken/Kagoshima/TU2-18_19/2021_H5N1
A/chicken/Ehime/TU12-2-16_17/2022_H5N1
A/chicken/Ehime/TU10-2-13/2022_H5N1
A/chicken/Ehime/TU11-2-24_25/2022_H5N1
A/duck/Hulu Sungai Utara/A0522064-06/2022_H5N1
A/mandarinduck/Korea/WA585/2021_H5N1
A/chicken/Aomori/TU9-41_42/2021_H5N1
A/chicken/Hyogo/TU4-45_46/2021_H5N1
A/quail/Korea/H526/2021_H5N1
A/teal/Miyazaki/211109-32/2021_H5N1
A/chicken/Kagoshima/TU13-3_4/2022_H5N1
A/duck/Chiba/TU6-19_20/2021_H5N1
A/Black Faced Spoonbill/Hong Kong/AFCD-HKU-21-17198.01012/2021_H5N1
A/chicken/Saitama/TU7-34_35/2021_H5N1
A/common buzzard/Kyoto/2601B013/2022_H5N1
A/Curlew/Hong Kong/AFCD-HKU-22-01095.01009/2022_H5N1
A/Cygnuscolumbianus/Hubei/53/2020_H5N8
A/Chlidoniashybrida/Hubei/55/2020_H5N8
A/Cygnuscolumbianus/Hubei/116/2020_H5N8
A/whooperswan/Shanxi/4-1/2020_H5N8
A/Cygnuscolumbianus/Hubei/52/2020_H5N8
A/Cygnuscolumbianus/Hubei/49/2020_H5N8
A/Cygnuscolumbianus/Hubei/56/2020_H5N8
A/Peregrine_Falcon/HongKong/AFCD-HKT-21-013-27/2021_H5N8
A/water/Nagano/SW0214/2021_H5N8
A/commonteal/Chiba/T42-F1/2021_H5N8
A/environment/Tokushima/T47-36/2021_H5N8
A/Cygnuscolumbianus/Hubei/51/2020_H5N8
A/mallard/Miyazaki/4501C607-c3/2021_H5N8
A/mallard/Miyazaki/4501C607-c1/2021_H5N8
A/chicken/Kagoshima/TU3-46_47/2021_H5N8
A/environment/Akita/TU1-49/2021_H5N8
A/chicken/Chiba/T49-18/2021_H5N8
A/chicken/Chiba/T46-11/2021_H5N8
A/chicken/Chiba/T48-17/2021_H5N8
A/environment/sample/China/TZ001/2021_H5N8
A/eurasian coot/Shandong/W5611/2020_H5N8
A/goose/China/1/2021_H5N8
A/goose/Hebei/HG12/2021_H5N8
A/turkey/Omsk/0003/2020_H5N8
A/Cygnuscolumbianus/Hubei/50/2020_H5N8
A/swan/France/21P006202/2021_H5N8
A/chicken/Nigeria/VRD21-53B_21VIR2288-5/2021_H5N8
A/chicken/Nigeria/VRD21-43_21VIR2288-4/2021_H5N8
A/chicken/Nigeria/VRD21-035B/21VIR2288-1/2021_H5N8
A/Mule_duck/France/20339/2020_H5N8
A/Mule_duck/France/20347/2020_H5N8
A/chicken/France/21P008169/2021_H5N8
A/chicken/France/21P007258/2021_H5N8
A/chicken/Kazakhstan/23/2020_H5N8
A/common pochard/Egypt/D11979C/2021_H5N8
A/duck/Egypt/BA19903OP/2021_H5N8
A/chicken/Egypt/F17230B/2019_H5N8
A/duck/Egypt/A16793/2019_H5N8
A/duck/Egypt/A16368/2018_H5N8
A/chicken/Egypt/N16732/2019_H5N8
A/duck/Hubei/SE128/2022_H5N1
A/chicken/Jiangxi/S40653/2021_H5N1
A/dairy_cow/Texas/24-008749-007/2024_H5N1
A/dairy_cow/Texas/24-008749-003/2024_H5N1
0.02

E) MP

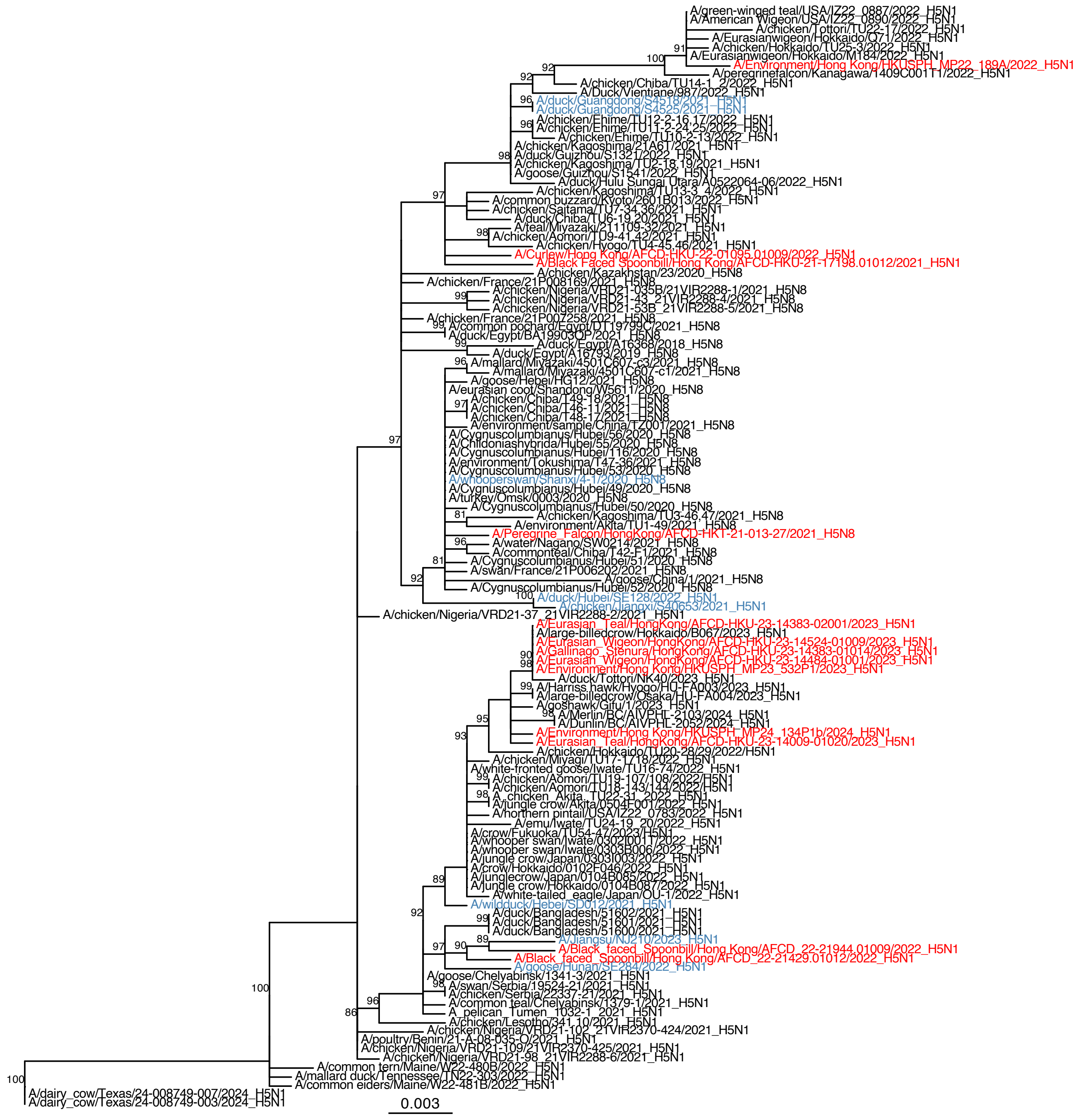

A/green-winged teal/USA/IZ22_0887/2022_H5N1
A/American Wigeon/USA/IZ22_0890/2022_H5N1
A/chicken/Tottori/TU22-17/2022_H5N1
A/Eurasianwigeon/Hokkaido/Q71/2022_H5N1
A/chicken/Hokkaido/TU25-3/2022_H5N1
A/Eurasianwigeon/Hokkaido/M184/2022_H5N1
A/Environment/Hong Kong/HKUSPH_MP22_189A/2022_H5N1
A/peregrinefalcon/Kanagawa/1409C001T1/2022_H5N1
A/chicken/Chiba/TU14-1_2/2022_H5N1
A/Duck/Vientiane/987/2022_H5N1
A/duck/Guangdong/S4518/2021_H5N1
A/duck/Guangdong/S4525/2021_H5N1
A/chicken/Ehime/TU12-2-16,17/2022_H5N1
A/chicken/Ehime/TU11-2-24,25/2022_H5N1
A/chicken/Ehime/TU10-2-13/2022_H5N1
A/chicken/Kagoshima/21A6T/2021_H5N1
A/duck/Guizhou/S1321/2022_H5N1
A/chicken/Kagoshima/TU2-18,19/2021_H5N1
A/goose/Guizhou/S1541/2022_H5N1
A/duck/Hulu Sungai Utara/A0522064-06/2022_H5N1
A/chicken/Kagoshima/TU13-3_4/2022_H5N1
A/common buzzard/Kyoto/2601B013/2022_H5N1
A/chicken/Saitama/TU7-34,36/2021_H5N1
A/duck/Chiba/TU6-19,20/2021_H5N1
A/teal/Miyazaki/211109-32/2021_H5N1
A/chicken/Aomori/TU9-41,42/2021_H5N1
A/chicken/Hyogo/TU4-45,46/2021_H5N1
A/Curlew/Hong Kong/AFCD-HKU-22-01095.01009/2022_H5N1
A/Black Faced Spoonbill/Hong Kong/AFCD-HKU-21-17198.01012/2021_H5N1
A/chicken/Kazakhstan/23/2020_H5N8
A/chicken/France/21P008169/2021_H5N8
A/chicken/Nigeria/VRD21-035B/21VIR2288-1/2021_H5N8
A/chicken/Nigeria/VRD21-43_21VIR2288-4/2021_H5N8
A/chicken/Nigeria/VRD21-53B_21VIR2288-5/2021_H5N8
A/chicken/France/21P007258/2021_H5N8
A/common pochard/Egypt/DT19799C/2021_H5N8
A/duck/Egypt/BA19903OP/2021_H5N8
A/duck/Egypt/A16368/2018_H5N8
A/duck/Egypt/A16793/2019_H5N8
A/mallard/Miyazaki/4501C607-c3/2021_H5N8
A/mallard/Miyazaki/4501C607-c1/2021_H5N8
A/goose/Hebei/HG12/2021_H5N8
A/eurasian coot/Shandong/W5611/2020_H5N8
A/chicken/Chiba/T49-18/2021_H5N8
A/chicken/Chiba/T46-11/2021_H5N8
A/chicken/Chiba/T48-17/2021_H5N8
A/environment/sample/China/TZ001/2021_H5N8
A/Cygnuscolumbianus/Hubei/56/2020_H5N8
A/Chlidoniashybrida/Hubei/55/2020_H5N8
A/Cygnuscolumbianus/Hubei/116/2020_H5N8
A/environment/Tokushima/T47-36/2021_H5N8
A/Cygnuscolumbianus/Hubei/53/2020_H5N8
A/whooperswan/Shanxi/4-1/2020_H5N8
A/Cygnuscolumbianus/Hubei/49/2020_H5N8
A/turkey/Omsk/0003/2020_H5N8
A/Cygnuscolumbianus/Hubei/50/2020_H5N8
A/chicken/Kagoshima/TU3-46,47/2021_H5N8
A/environment/Akita/TU1-49/2021_H5N8
A/Peregrine_Falcon/HongKong/AFCD-HKT-21-013-27/2021_H5N8
A/water/Nagano/SW0214/2021_H5N8
A/commonteal/Chiba/T42-F1/2021_H5N8
A/Cygnuscolumbianus/Hubei/51/2020_H5N8
A/swan/France/21P006202/2021_H5N8
A/goose/China/1/2021_H5N8
A/Cygnuscolumbianus/Hubei/52/2020_H5N8
A/duck/Hubei/SE128/2022_H5N1
A/chicken/Jiangxi/S40653/2021_H5N1
A/chicken/Nigeria/VRD21-37_21VIR2288-2/2021_H5N1
A/Eurasian_Teal/HongKong/AFCD-HKU-23-14383-02001/2023_H5N1
A/large-billedcrow/Hokkaido/B067/2023_H5N1
A/Eurasian_Wigeon/HongKong/AFCD-HKU-23-14524-01009/2023_H5N1
A/Gallinago_Stenura/HongKong/AFCD-HKU-23-14383-01014/2023_H5N1
A/Eurasian_Wigeon/HongKong/AFCD-HKU-23-14484-01001/2023_H5N1
A/Environment/Hong Kong/HKUSPH_MP23_532P1/2023_H5N1
A/duck/Tottori/NK40/2023_H5N1
A/Harriss hawk/Hyogo/HU-FA003/2023_H5N1
A/large-billedcrow/Osaka/HU-FA004/2023_H5N1
A/goshawk/Gifu/1/2023_H5N1
A/Merlin/BC/AIVPHL-2103/2024_H5N1
A/Dunlin/BC/AIVPHL-2052/2024_H5N1
A/Environment/Hong Kong/HKUSPH_MP24_134P1b/2024_H5N1
A/Eurasian_Teal/HongKong/AFCD-HKU-23-14009-01020/2023_H5N1
A/chicken/Hokkaido/TU20-28/29/2022/H5N1
A/chicken/Miyagi/TU17-1718/2022_H5N1
A/white-fronted goose/Iwate/TU16-74/2022_H5N1
A/chicken/Aomori/TU19-107/108/2022/H5N1
A/chicken/Aomori/TU18-143/144/2022/H5N1
A_chicken_Akita_TU22-31_2022_H5N1
A/jungle crow/Akita/0504F001/2022_H5N1
A/northern pintail/USA/IZ22_0783/2022_H5N1
A/emu/Iwate/TU24-19_20/2022_H5N1
A/crow/Fukuoka/TU54-47/2023/H5N1
A/whooper swan/Iwate/0302I001T/2022_H5N1
A/whooper swan/Iwate/0303B006/2022_H5N1
A/jungle crow/Japan/0303I003/2022_H5N1
A/crow/Hokkaido/0102F046/2022_H5N1
A/junglecrow/Japan/0104B085/2022_H5N1
A/jungle crow/Hokkaido/0104B087/2022_H5N1
A/white-tailed_eagle/Japan/OU-1/2022_H5N1
A/wildduck/Hebei/SD012/2021_H5N1
A/duck/Bangladesh/51602/2021_H5N1
A/duck/Bangladesh/51601/2021_H5N1
A/duck/Bangladesh/51600/2021_H5N1
A/Jiangsu/NJ210/2023_H5N1
A/Black_faced_Spoonbill/Hong Kong/AFCD_22-21944.01009/2022_H5N1
A/Black_faced_Spoonbill/Hong Kong/AFCD_22-21429.01012/2022_H5N1
A/goose/Hunan/SE284/2022_H5N1
A/goose/Chelyabinsk/1341-3/2021_H5N1
A/swan/Serbia/19524-21/2021_H5N1
A/chicken/Serbia/22337-21/2021_H5N1
A/common teal/Chelyabinsk/1379-1/2021_H5N1
A_pelican_Tumen_1032-1_2021_H5N1
A/chicken/Lesotho/341.10/2021_H5N1
A/chicken/Nigeria/VRD21-102_21VIR2370-424/2021_H5N1
A/poultry/Benin/21-A-08-035-O/2021_H5N1
A/chicken/Nigeria/VRD21-109/21VIR2370-425/2021_H5N1
A/chicken/Nigeria/VRD21-98_21VIR2288-6/2021_H5N1
A/common tern/Maine/W22-480B/2022_H5N1
A/mallard duck/Tennessee/TN22-303/2022_H5N1
A/common eiders/Maine/W22-481B/2022_H5N1
A/dairy_cow/Texas/24-008749-007/2024_H5N1
A/dairy_cow/Texas/24-008749-003/2024_H5N1
0.003

F) NS

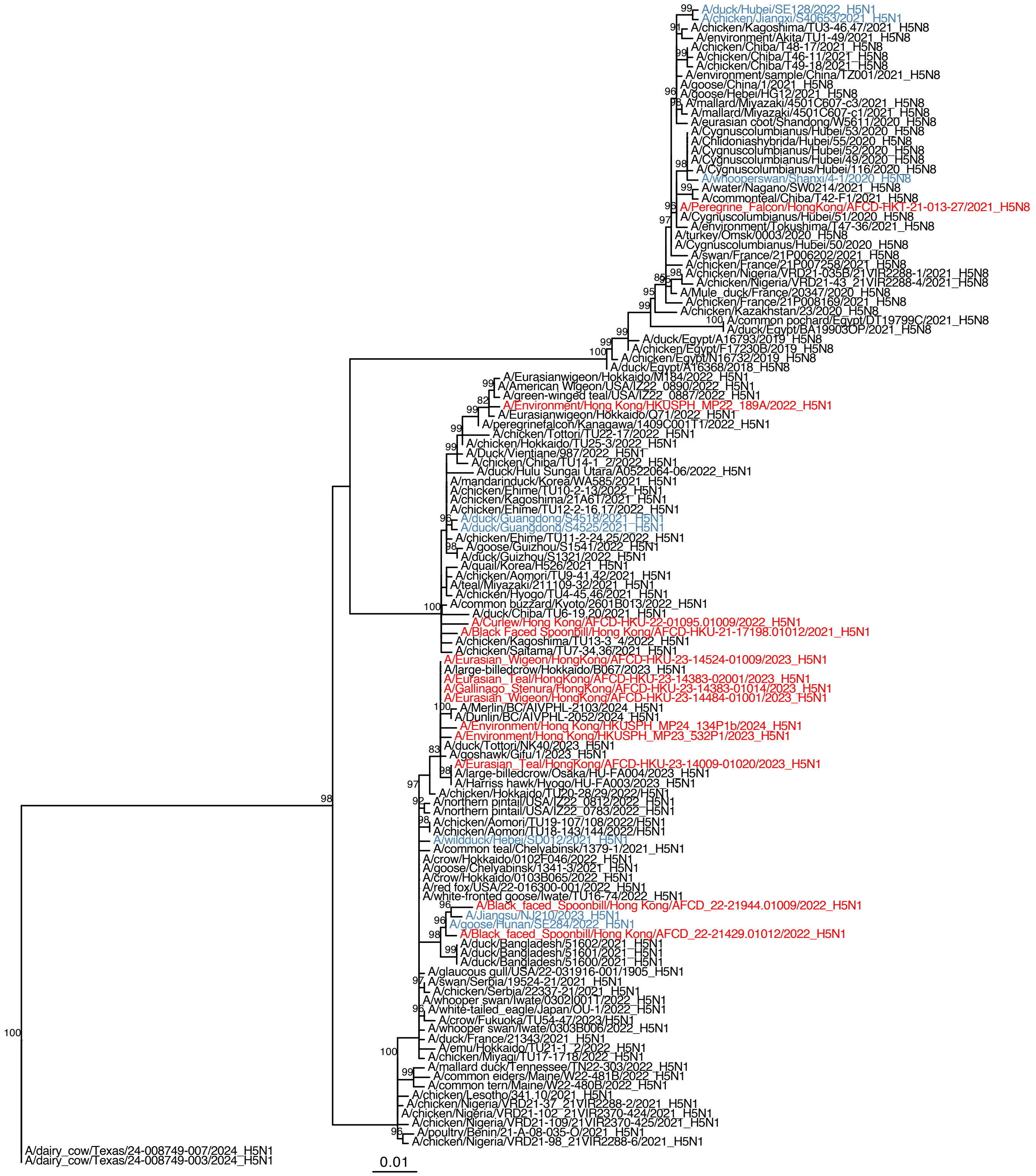

99
A/duck/Hubei/SE128/2022_H5N1
A/chicken/Jiangxi/S40653/2021_H5N1
91
A/chicken/Kagoshima/TU3-46,47/2021_H5N8
A/environment/Akita/TU1-49/2021_H5N8
A/chicken/Chiba/T48-17/2021_H5N8
99
A/chicken/Chiba/T46-11/2021_H5N8
A/chicken/Chiba/T49-18/2021_H5N8
A/environment/sample/China/TZ001/2021_H5N8
A/goose/China/1/2021_H5N8
96
A/goose/Hebei/HG12/2021_H5N8
98
A/mallard/Miyazaki/4501C607-c3/2021_H5N8
A/mallard/Miyazaki/4501C607-c1/2021_H5N8
A/eurasian coot/Shandong/W5611/2020_H5N8
A/Cygnuscolumbianus/Hubei/53/2020_H5N8
A/Chlidoniashybrida/Hubei/55/2020_H5N8
A/Cygnuscolumbianus/Hubei/52/2020_H5N8
A/Cygnuscolumbianus/Hubei/49/2020_H5N8
98
A/Cygnuscolumbianus/Hubei/116/2020_H5N8
A/whooperswan/Shanxi/4-1/2020_H5N8
99
A/water/Nagano/SW0214/2021_H5N8
A/commonteal/Chiba/T42-F1/2021_H5N8
96
A/Peregrine_Falcon/HongKong/AFCD-HKT-21-013-27/2021_H5N8
97
A/Cygnuscolumbianus/Hubei/51/2020_H5N8
A/environment/Tokushima/T47-36/2021_H5N8
A/turkey/Omsk/0003/2020_H5N8
A/Cygnuscolumbianus/Hubei/50/2020_H5N8
A/swan/France/21P006202/2021_H5N8
A/chicken/France/21P007258/2021_H5N8
98
A/chicken/Nigeria/VRD21-035B/21VIR2288-1/2021_H5N8
85
95
A/chicken/Nigeria/VRD21-43_21VIR2288-4/2021_H5N8
95
A/Mule_duck/France/20347/2020_H5N8
A/chicken/France/21P008169/2021_H5N8
99
A/chicken/Kazakhstan/23/2020_H5N8
100
A/common pochard/Egypt/DT19799C/2021_H5N8
A/duck/Egypt/BA19903OP/2021_H5N8
99
99
A/duck/Egypt/A16793/2019_H5N8
A/chicken/Egypt/F17230B/2019_H5N8
100
A/chicken/Egypt/N16732/2019_H5N8
A/duck/Egypt/A16368/2018_H5N8
A/Eurasianwigeon/Hokkaido/M184/2022_H5N1
99
A/American Wigeon/USA/IZ22_0890/2022_H5N1
A/green-winged teal/USA/IZ22_0887/2022_H5N1
82
A/Environment/Hong Kong/HKUSPH_MP22_189A/2022_H5N1
99
A/Eurasianwigeon/Hokkaido/Q71/2022_H5N1
A/peregrinefalcon/Kanagawa/1409C001T1/2022_H5N1
99
A/chicken/Tottori/TU22-17/2022_H5N1
99
A/chicken/Hokkaido/TU25-3/2022_H5N1
A/Duck/Vientiane/987/2022_H5N1
A/chicken/Chiba/TU14-1_2/2022_H5N1
A/duck/Hulu Sungai Utara/A0522064-06/2022_H5N1
A/mandarinduck/Korea/WA585/2021_H5N1
A/chicken/Ehime/TU10-2-13/2022_H5N1
A/chicken/Kagoshima/21A61/2021_H5N1
A/chicken/Ehime/TU12-2-16,17/2022_H5N1
96
A/duck/Guangdong/S4518/2021_H5N1
A/duck/Guangdong/S4525/2021_H5N1
A/chicken/Ehime/TU11-2-24,25/2022_H5N1
98
A/goose/Guizhou/S1541/2022_H5N1
A/duck/Guizhou/S1321/2022_H5N1
A/quail/Korea/H526/2021_H5N1
A/chicken/Aomori/TU9-41,42/2021_H5N1
A/teal/Miyazaki/211109-32/2021_H5N1
A/chicken/Hyogo/TU4-45,46/2021_H5N1
100
A/common buzzard/Kyoto/2601B013/2022_H5N1
A/duck/Chiba/TU6-19,20/2021_H5N1
A/Curlew/Hong Kong/AFCD-HKU-22-01095.01009/2022_H5N1
A/Black Faced Spoonbill/Hong Kong/AFCD-HKU-21-17198.01012/2021_H5N1
A/chicken/Kagoshima/TU13-3_4/2022_H5N1
A/chicken/Saitama/TU7-34,36/2021_H5N1
A/Eurasian_Wigeon/HongKong/AFCD-HKU-23-14524-01009/2023_H5N1
A/large-billedcrow/Hokkaido/B067/2023_H5N1
A/Eurasian_Teal/HongKong/AFCD-HKU-23-14383-02001/2023_H5N1
A/Gallinago_Stenura/HongKong/AFCD-HKU-23-14383-01014/2023_H5N1
A/Eurasian_Wigeon/HongKong/AFCD-HKU-23-14484-01001/2023_H5N1
100
A/Merlin/BC/AIVPHL-2103/2024_H5N1
A/Dunlin/BC/AIVPHL-2052/2024_H5N1
A/Environment/Hong Kong/HKUSPH_MP24_134P1b/2024_H5N1
A/Environment/Hong Kong/HKUSPH_MP23_532P1/2023_H5N1
83
A/duck/Tottori/NK40/2023_H5N1
A/goshawk/Gifu/1/2023_H5N1
A/Eurasian_Teal/HongKong/AFCD-HKU-23-14009-01020/2023_H5N1
98
A/large-billedcrow/Osaka/HU-FA004/2023_H5N1
97
A/Harriss hawk/Hyogo/HU-FA003/2023_H5N1
98
A/chicken/Hokkaido/TU20-28/29/2022/H5N1
92
A/northern pintail/USA/IZ22_0812/2022_H5N1
A/northern pintail/USA/IZ22_0783/2022_H5N1
98
A/chicken/Aomori/TU19-107/108/2022/H5N1
A/chicken/Aomori/TU18-143/144/2022/H5N1
A/wildduck/Hebei/SD012/2021_H5N1
A/common teal/Chelyabinsk/1379-1/2021_H5N1
A/crow/Hokkaido/0102F046/2022_H5N1
A/goose/Chelyabinsk/1341-3/2021_H5N1
A/crow/Hokkaido/0103B065/2022_H5N1
A/red fox/USA/22-016300-001/2022_H5N1
A/white-fronted goose/Iwate/TU16-74/2022_H5N1
96
A/Black_faced_Spoonbill/Hong Kong/AFCD_22-21944.01009/2022_H5N1
A/Jiangsu/NJ210/2023_H5N1
96
A/goose/Hunan/SE284/2022_H5N1
98
A/Black_faced_Spoonbill/Hong Kong/AFCD_22-21429.01012/2022_H5N1
A/duck/Bangladesh/51602/2021_H5N1
99
A/duck/Bangladesh/51601/2021_H5N1
A/duck/Bangladesh/51600/2021_H5N1
A/glaucous gull/USA/22-031916-001/1905_H5N1
97
A/swan/Serbia/19524-21/2021_H5N1
A/chicken/Serbia/22337-21/2021_H5N1
A/whooper swan/Iwate/0302I001T/2022_H5N1
96
A/white-tailed_eagle/Japan/OU-1/2022_H5N1
A/crow/Fukuoka/TU54-47/2023/H5N1
A/whooper swan/Iwate/0303B006/2022_H5N1
A/duck/France/21343/2021_H5N1
100
A/emu/Hokkaido/TU21-1_2/2022_H5N1
A/chicken/Miyagi/TU17-17/18/2022_H5N1
A/mallard duck/Tennessee/TN22-303/2022_H5N1
99
A/common eiders/Maine/W22-481B/2022_H5N1
A/common tern/Maine/W22-480B/2022_H5N1
A/chicken/Lesotho/341.10/2021_H5N1
A/chicken/Nigeria/VRD21-37_21VIR2288-2/2021_H5N1
A/chicken/Nigeria/VRD21-102_21VIR2370-424/2021_H5N1
A/chicken/Nigeria/VRD21-109/21VIR2370-425/2021_H5N1
96
A/poultry/Benin/21-A-08-035-O/2021_H5N1
A/chicken/Nigeria/VRD21-98_21VIR2288-6/2021_H5N1
98
100
A/dairy_cow/Texas/24-008749-007/2024_H5N1
A/dairy_cow/Texas/24-008749-003/2024_H5N1
0.01

Table S1 Presents the genome identity between samples collected from Hong Kong and A/Jiangsu/NJ210/2023

HA	A/Black_Faced_Spoonbill/Hong Kong/AFCD-HKU-22-21944-01009/2022	A/Curlew/Hong Kong/AFCD-HKU-22-01095.01009/2022	A/Black Faced Spoonbill/Hong Kong/AFCD-HKU-22-21429-01012/2022	A/Environment/Hong Kong/HKUSPH_MP24_134P1b/2024	A/Black Faced Spoonbill/Hong Kong/AFCD-HKU-21-17198.01012/2021	A_Eurasian_Teal_HongKong_AFCD-HKU-23-14009-01020_2023	A/Environment/Hong Kong/HKUSPH_MP23_532P1/2023	A/Environment/Hong Kong/HKUSPH_MP22_189A/2022	A/Gallinago Stenura/Hong Kong/AFCD-HKU-23-14383-01014/2023	A/Peregrine Falcon/Hong Kong/AFCD-HKU-21-013-27/2021	A/Eurasian Teal/Hong Kong/AFCD-HKU-23-14383-02001/2023	A/Jiangsu/NJ210/2023	A/Eurasian Wigeon/Hong Kong/AFCD-HKU-23-14524-01009/2023	A/Eurasian Wigeon/Hong Kong/AFCD-HKU-23-14484-01001/2023
A/Black_Faced_Spoonbill/Hong Kong/AFCD-HKU-22-21944-01009/2022	1.000	0.975	0.997	0.984	0.974	0.986	0.986	0.972	0.985	0.984	0.985	0.996	0.986	0.986
A/Curlew/Hong Kong/AFCD-HKU-22-01095.01009/2022	0.975	1.000	0.973	0.977	0.991	0.979	0.978	0.986	0.978	0.982	0.976	0.973	0.978	0.978
A/Black Faced Spoonbill/Hong Kong/AFCD-HKU-22-21429-01012/2022	0.997	0.973	1.000	0.982	0.972	0.985	0.984	0.970	0.984	0.982	0.983	0.994	0.984	0.984
A/Environment/Hong Kong/HKUSPH_MP24_134P1b/2024	0.984	0.977	0.982	1.000	0.975	0.996	0.996	0.973	0.996	0.986	0.994	0.982	0.996	0.996
A/Black Faced Spoonbill/Hong Kong/AFCD-HKU-21-17198.01012/2021	0.974	0.991	0.972	0.975	1.000	0.978	0.977	0.985	0.976	0.981	0.975	0.972	0.977	0.977
A_Eurasian_Teal_HongKong_AFCD-HKU-23-14009-01020_2023	0.986	0.979	0.985	0.996	0.978	1.000	0.998	0.976	0.998	0.989	0.996	0.985	0.998	0.998
A/Environment/Hong Kong/HKUSPH_MP23_532P1/2023	0.986	0.978	0.984	0.996	0.977	0.998	1.000	0.975	0.998	0.988	0.996	0.984	0.999	0.999
A/Environment/Hong Kong/HKUSPH_MP22_189A/2022	0.972	0.986	0.970	0.973	0.985	0.976	0.975	1.000	0.975	0.979	0.973	0.970	0.975	0.975
A/Gallinago Stenura/Hong Kong/AFCD-HKU-23-14383-01014/2023	0.985	0.978	0.984	0.996	0.976	0.998	0.998	0.975	1.000	0.988	0.997	0.984	0.999	0.999
A/Peregrine Falcon/Hong Kong/AFCD-HKU-21-013-27/2021	0.984	0.982	0.982	0.986	0.981	0.989	0.988	0.979	0.988	1.000	0.986	0.982	0.988	0.988
A/Eurasian Teal/Hong Kong/AFCD-HKU-23-14383-02001/2023	0.985	0.976	0.983	0.994	0.975	0.996	0.996	0.973	0.997	0.986	1.000	0.983	0.998	0.998
A/Jiangsu/NJ210/2023	0.996	0.973	0.994	0.982	0.972	0.985	0.984	0.970	0.984	0.982	0.983	1.000	0.984	0.984
A/Eurasian Wigeon/Hong Kong/AFCD-HKU-23-14524-01009/2023	0.986	0.978	0.984	0.996	0.977	0.998	0.999	0.975	0.999	0.988	0.998	0.984	1.000	1.000
A/Eurasian Wigeon/Hong Kong/AFCD-HKU-23-14484-01001/2023	0.986	0.978	0.984	0.996	0.977	0.998	0.999	0.975	0.999	0.988	0.998	0.984	1.000	1.000

N1	A/Gallinago Stenura/Hong Kong/AFCD-HKU-23-14383-01014/2023	A/Eurasian Wigeon/Hong Kong/AFCD-HKU-23-14484-01001/2023	A/Eurasian Wigeon/Hong Kong/AFCD-HKU-23-14524-01009/2023	A/Curlew/Hong Kong/AFCD-HKU-22-01095.01009/2022	A/Environment/Hong Kong/HKUSPH_MP22_189A/2022	A/Eurasian Teal/Hong Kong/AFCD-HKU-23-14383-02001/2023	A_Eurasian_Teal_HongKong_AFCD-HKU-23-14009-01020_2023	A/Black Faced Spoonbill/Hong Kong/AFCD-HKU-21-17198.01012/2021	A/Environment/Hong Kong/HKUSPH_MP23_532P1/2023	A/Environment/Hong Kong/HKUSPH_MP24_134P1b/2024	A/Black Faced Spoonbill/Hong Kong/AFCD-HKU-22-21429-01012/2022	A/Jiangsu/NJ210/2023	A/Black_Faced_Spoonbill/Hong Kong/AFCD-HKU-22-21944-01009/2022	
A/Gallinago Stenura/Hong Kong/AFCD-HKU-23-14383-01014/2023	1.000	1.000	0.999	0.967	0.967	0.999	0.997	0.968	0.997	0.994	0.986	0.984	0.984	
A/Eurasian Wigeon/Hong Kong/AFCD-HKU-23-14484-01001/2023	1.000	1.000	0.999	0.967	0.967	0.999	0.997	0.968	0.997	0.994	0.986	0.984	0.984	
A/Eurasian Wigeon/Hong Kong/AFCD-HKU-23-14524-01009/2023	0.999	0.999	1.000	0.966	0.967	0.999	0.996	0.967	0.996	0.994	0.985	0.983	0.984	
A/Curlew/Hong Kong/AFCD-HKU-22-01095.01009/2022	0.967	0.967	0.966	1.000	0.991	0.966	0.967	0.991	0.967	0.964	0.967	0.965	0.966	
A/Environment/Hong Kong/HKUSPH_MP22_189A/2022	0.967	0.967	0.967	0.991	1.000	0.967	0.967	0.992	0.967	0.965	0.968	0.966	0.967	
A/Eurasian Teal/Hong Kong/AFCD-HKU-23-14383-02001/2023	0.999	0.999	0.999	0.966	0.967	1.000	0.996	0.967	0.996	0.994	0.985	0.983	0.984	
A_Eurasian_Teal_HongKong_AFCD-HKU-23-14009-01020_2023	0.997	0.997	0.996	0.967	0.967	0.996	1.000	0.968	0.996	0.994	0.986	0.984	0.984	
A/Black Faced Spoonbill/Hong Kong/AFCD-HKU-21-17198.01012/2021	0.968	0.968	0.967	0.991	0.992	0.967	0.968	1.000	0.968	0.965	0.969	0.966	0.967	
A/Environment/Hong Kong/HKUSPH_MP23_532P1/2023	0.997	0.997	0.996	0.967	0.967	0.996	0.996	0.968	1.000	0.993	0.984	0.982	0.983	
A/Environment/Hong Kong/HKUSPH_MP24_134P1b/2024	0.994	0.994	0.994	0.964	0.965	0.994	0.994	0.965	0.993	1.000	0.983	0.981	0.982	
A/Black Faced Spoonbill/Hong Kong/AFCD-HKU-22-21429-01012/2022	0.986	0.986	0.985	0.967	0.968	0.985	0.986	0.969	0.984	0.983	1.000	0.995	0.995	
A/Jiangsu/NJ210/2023	0.984	0.984	0.983	0.965	0.966	0.983	0.984	0.966	0.982	0.981	0.995	1.000	0.993	
A/Black_Faced_Spoonbill/Hong Kong/AFCD-HKU-22-21944-01009/2022	0.984	0.984	0.984	0.966	0.967	0.984	0.984	0.967	0.983	0.982	0.995	0.993	1.000	

PB2	A/Curlew/Hong Kong/AFCD-HKU-22-01095.01009/2022	A/Environment/Hong Kong/HKUSPH_MP24_134P1b/2024	A/Black Faced Spoonbill/Hong Kong/AFCD-HKU-22-21429-01012/2022	A/Eurasian Wigeon/Hong Kong/AFCD-HKU-23-14524-01009/2023	A/Eurasian Wigeon/Hong Kong/AFCD-HKU-23-14484-01001/2023	A/Black_Faced_Spoonbill/Hong Kong/AFCD-HKU-22-21944-01009/2022	A/Gallinago Stenura/Hong Kong/AFCD-HKU-23-14383-01014/2023	A/Environment/Hong Kong/HKUSPH_MP22_189A/2022	A/Peregrine Falcon/Hong Kong/AFCD-HKU-21-013-27/2021	A/Black Faced Spoonbill/Hong Kong/AFCD-HKU-21-17198.01012/2021	A/Eurasian Teal/Hong Kong/AFCD-HKU-23-14383-02001/2023	A/Environment/Hong Kong/HKUSPH_MP23_532P1/2023	A_Eurasian_Teal_HongKong_AFCD-HKU-23-14009-01020_2023	A/Jiangsu/NJ210/2023
A/Curlew/Hong Kong/AFCD-HKU-22-01095.01009/2022	1.000	0.912	0.938	0.911	0.911	0.939	0.911	0.991	0.933	0.996	0.911	0.912	0.913	0.935
A/Environment/Hong Kong/HKUSPH_MP24_134P1b/2024	0.912	1.000	0.904	0.991	0.991	0.907	0.991	0.912	0.896	0.912	0.992	0.993	0.991	0.904
A/Black Faced Spoonbill/Hong Kong/AFCD-HKU-22-21429-01012/2022	0.938	0.904	1.000	0.905	0.905	0.992	0.905	0.939	0.954	0.939	0.905	0.907	0.906	0.989
A/Eurasian Wigeon/Hong Kong/AFCD-HKU-23-14524-01009/2023	0.911	0.991	0.905	1.000	1.000	0.907	1.000	0.910	0.898	0.911	1.000	0.998	0.994	0.904
A/Eurasian Wigeon/Hong Kong/AFCD-HKU-23-14484-01001/2023	0.911	0.991	0.905	1.000	1.000	0.907	1.000	0.910	0.898	0.911	1.000	0.998	0.994	0.904
A/Black_Faced_Spoonbill/Hong Kong/AFCD-HKU-22-21944-01009/2022	0.939	0.907	0.992	0.907	0.907	1.000	0.907	0.939	0.956	0.940	0.907	0.909	0.908	0.994
A/Gallinago Stenura/Hong Kong/AFCD-HKU-23-14383-01014/2023	0.911	0.991	0.905	1.000	1.000	0.907	1.000	0.910	0.898	0.911	1.000	0.998	0.994	0.904
A/Environment/Hong Kong/HKUSPH_MP22_189A/2022	0.991	0.912	0.939	0.910	0.910	0.939	0.910	1.000	0.932	0.992	0.910	0.911	0.912	0.935
A/Peregrine Falcon/Hong Kong/AFCD-HKU-21-013-27/2021	0.933	0.896	0.954	0.898	0.898	0.956	0.898	0.932	1.000	0.934	0.898	0.900	0.898	0.953
A/Black Faced Spoonbill/Hong Kong/AFCD-HKU-21-17198.01012/2021	0.996	0.912	0.939	0.911	0.911	0.940	0.911	0.992	0.934	1.000	0.911	0.912	0.913	0.936
A/Eurasian Teal/Hong Kong/AFCD-HKU-23-14383-02001/2023	0.911	0.992	0.905	1.000	1.000	0.907	1.000	0.910	0.898	0.911	1.000	0.998	0.995	0.905
A/Environment/Hong Kong/HKUSPH_MP23_532P1/2023	0.912	0.993	0.907	0.998	0.998	0.909	0.998	0.911	0.900	0.912	0.998	1.000	0.996	0.907
A_Eurasian_Teal_HongKong_AFCD-HKU-23-14009-01020_2023	0.913	0.991	0.906	0.994	0.994	0.908	0.994	0.912	0.898	0.913	0.995	0.996	1.000	0.906
A/Jiangsu/NJ210/2023	0.935	0.904	0.989	0.904	0.904	0.994	0.904	0.935	0.953	0.936	0.905	0.907	0.906	1.000

PB1	A_Eurasian_Teal_HongKong_AFCD-HKU-23-14009-01020_2023	A/Eurasian Wigeon/Hong Kong/AFCD-HKU-23-14484-01001/2023	A/Eurasian Wigeon/Hong Kong/AFCD-HKU-23-14524-01009/2023	A/Gallinago Stenura/Hong Kong/AFCD-HKU-23-14383-01014/2023	A/Black Faced Spoonbill/Hong Kong/AFCD-HKU-22-21429-01012/2022	A/Curlew/Hong Kong/AFCD-HKU-22-01095.01009/2022	A/Environment/Hong Kong/HKUSPH_MP22_189A/2022	A/Environment/Hong Kong/HKUSPH_MP23_532P1/2023	A/Black Faced Spoonbill/Hong Kong/AFCD-HKU-21-17198.01012/2021	A/Eurasian Teal/Hong Kong/AFCD-HKU-23-14383-02001/2023	A/Environment/Hong Kong/HKUSPH_MP24_134P1b/2024	A/Jiangsu/NJ210/2023	A/Black_Faced_Spoonbill/Hong Kong/AFCD-HKU-22-21944-01009/2022	A/Peregrine Falcon/Hong Kong/AFCD-HKU-21-013-27/2021
A_Eurasian_Teal_HongKong_AFCD-HKU-23-14009-01020_2023	1.000	0.996	0.996	0.996	0.988	0.927	0.924	0.997	0.927	0.996	0.996	0.989	0.989	0.918
A/Eurasian Wigeon/Hong Kong/AFCD-HKU-23-14484-01001/2023	0.996	1.000	1.000	1.000	0.988	0.927	0.924	0.999	0.927	1.000	0.996	0.989	0.989	0.918
A/Eurasian Wigeon/Hong Kong/AFCD-HKU-23-14524-01009/2023	0.996	1.000	1.000	1.000	0.988	0.927	0.924	0.999	0.927	1.000	0.996	0.989	0.989	0.918
A/Gallinago Stenura/Hong Kong/AFCD-HKU-23-14383-01014/2023	0.996	1.000	1.000	1.000	0.988	0.927	0.924	0.999	0.927	1.000	0.996	0.989	0.989	0.918
A/Black Faced Spoonbill/Hong Kong/AFCD-HKU-22-21429-01012/2022	0.988	0.988	0.988	0.988	1.000	0.926	0.923	0.989	0.926	0.988	0.987	0.995	0.994	0.916
A/Curlew/Hong Kong/AFCD-HKU-22-01095.01009/2022	0.927	0.927	0.927	0.927	0.926	1.000	0.989	0.928	0.996	0.927	0.927	0.927	0.926	0.941
A/Environment/Hong Kong/HKUSPH_MP22_189A/2022	0.924	0.924	0.924	0.924	0.923	0.989	1.000	0.925	0.987	0.924	0.924	0.923	0.923	0.934
A/Environment/Hong Kong/HKUSPH_MP23_532P1/2023	0.997	0.999	0.999	0.999	0.989	0.928	0.925	1.000	0.928	0.999	0.996	0.990	0.989	0.919
A/Black Faced Spoonbill/Hong Kong/AFCD-HKU-21-17198.01012/2021	0.927	0.927	0.927	0.927	0.926	0.996	0.987	0.928	1.000	0.927	0.927	0.926	0.926	0.939
A/Eurasian Teal/Hong Kong/AFCD-HKU-23-14383-02001/2023	0.996	1.000	1.000	1.000	0.988	0.927	0.924	0.999	0.927	1.000	0.996	0.989	0.989	0.918
A/Environment/Hong Kong/HKUSPH_MP24_134P1b/2024	0.996	0.996	0.996	0.996	0.987	0.927	0.924	0.996	0.927	0.996	1.000	0.988	0.988	0.918
A/Jiangsu/NJ210/2023	0.989	0.989	0.989	0.989	0.995	0.927	0.923	0.990	0.926	0.989	0.988	1.000	0.996	0.917
A/Black_Faced_Spoonbill/Hong Kong/AFCD-HKU-22-21944-01009/2022	0.989	0.989	0.989	0.989	0.994	0.926	0.923	0.989	0.926	0.989	0.988	0.996	1.000	0.916
A/Peregrine Falcon/Hong Kong/AFCD-HKU-21-013-27/2021	0.918	0.918	0.918	0.918	0.916	0.941	0.934	0.919	0.939	0.918	0.918	0.917	0.916	1.000

PA	A/Environment/Hong Kong/HKUSPH_MP22_189A/2022	A/Curlew/Hong Kong/AFCD-HKU-22-01095.01009/2022	A/Jiangsu/NJ210/2023	A/Black Faced Spoonbill/Hong Kong/AFCD-HKU-21-17198.01012/2021	A/Environment/Hong Kong/HKUSPH_MP24_134P1b/2024	A/Black_Faced_Spoonbill/Hong Kong/AFCD-HKU-22-21944-01009/2022	A/Peregrine Falcon/Hong Kong/AFCD-HKU-21-013-27/2021	A/Eurasian Wigeon/Hong Kong/AFCD-HKU-23-14484-01001/2023	A/Eurasian Wigeon/Hong Kong/AFCD-HKU-23-14524-01009/2023	A/Black Faced Spoonbill/Hong Kong/AFCD-HKU-22-21429-01012/2022	A/Eurasian Teal/Hong Kong/AFCD-HKU-23-14383-02001/2023	A_Eurasian_Teal_HongKong_AFCD-HKU-23-14009-01020_2023	A/Gallinago Stenura/Hong Kong/AFCD-HKU-23-14383-01014/2023	A/Environment/Hong Kong/HKUSPH_MP23_532P1/2023
A/Environment/Hong Kong/HKUSPH_MP22_189A/2022	1.000	0.986	0.966	0.988	0.966	0.965	0.940	0.967	0.967	0.965	0.967	0.968	0.967	0.967
A/Curlew/Hong Kong/AFCD-HKU-22-01095.01009/2022	0.986	1.000	0.969	0.993	0.970	0.968	0.945	0.971	0.971	0.969	0.971	0.973	0.971	0.971
A/Jiangsu/NJ210/2023	0.966	0.969	1.000	0.972	0.985	0.994	0.943	0.986	0.986	0.994	0.986	0.987	0.986	0.986
A/Black Faced Spoonbill/Hong Kong/AFCD-HKU-21-17198.01012/2021	0.988	0.993	0.972	1.000	0.972	0.971	0.947	0.973	0.973	0.971	0.973	0.974	0.973	0.973
A/Environment/Hong Kong/HKUSPH_MP24_134P1b/2024	0.966	0.970	0.985	0.972	1.000	0.985	0.942	0.995	0.995	0.984	0.995	0.995	0.995	0.994
A/Black_Faced_Spoonbill/Hong Kong/AFCD-HKU-22-21944-01009/2022	0.965	0.968	0.994	0.971	0.985	1.000	0.941	0.986	0.986	0.994	0.986	0.987	0.986	0.986
A/Peregrine Falcon/Hong Kong/AFCD-HKU-21-013-27/2021	0.940	0.945	0.943	0.947	0.942	0.941	1.000	0.945	0.945	0.941	0.945	0.943	0.945	0.943
A/Eurasian Wigeon/Hong Kong/AFCD-HKU-23-14484-01001/2023	0.967	0.971	0.986	0.973	0.995	0.986	0.945	1.000	1.000	0.986	1.000	0.997	1.000	0.999
A/Eurasian Wigeon/Hong Kong/AFCD-HKU-23-14524-01009/2023	0.967	0.971	0.986	0.973	0.995	0.986	0.945	1.000	1.000	0.986	1.000	0.997	1.000	0.999
A/Black Faced Spoonbill/Hong Kong/AFCD-HKU-22-21429-01012/2022	0.965	0.969	0.994	0.971	0.984	0.994	0.941	0.986	0.986	1.000	0.986	0.986	0.986	0.985
A/Eurasian Teal/Hong Kong/AFCD-HKU-23-14383-02001/2023	0.967	0.971	0.986	0.973	0.995	0.986	0.945	1.000	1.000	0.986	1.000	0.997	1.000	0.999
A_Eurasian_Teal_HongKong_AFCD-HKU-23-14009-01020_2023	0.968	0.973	0.987	0.974	0.995	0.987	0.943	0.997	0.997	0.986	0.997	1.000	0.997	0.996
A/Gallinago Stenura/Hong Kong/AFCD-HKU-23-14383-01014/2023	0.967	0.971	0.986	0.973	0.995	0.986	0.945	1.000	1.000	0.986	1.000	0.997	1.000	0.999
A/Environment/Hong Kong/HKUSPH_MP23_532P1/2023	0.967	0.971	0.986	0.973	0.994	0.986	0.943	0.999	0.999	0.985	0.999	0.996	0.999	1.000

NP	A/Environment/Hong Kong/HKUSPH_MP23_532P1/2023	A/Eurasian Teal/Hong Kong/AFCD-HKU-23-14383-02001/2023	A/Environment/Hong Kong/HKUSPH_MP24_134P1b/2024	A/Black_Faced_Spoonbill/Hong Kong/AFCD-HKU-22-21944-01009/2022	A_Eurasian_Teal_HongKong_AFCD-HKU-23-14009-01020_2023	A/Curlew/Hong Kong/AFCD-HKU-22-01095.01009/2022	A/Black Faced Spoonbill/Hong Kong/AFCD-HKU-22-21429-01012/2022	A/Black Faced Spoonbill/Hong Kong/AFCD-HKU-21-17198.01012/2021	A/Gallinago Stenura/Hong Kong/AFCD-HKU-23-14383-01014/2023	A/Peregrine Falcon/Hong Kong/AFCD-HKU-21-013-27/2021	A/Eurasian Wigeon/Hong Kong/AFCD-HKU-23-14484-01001/2023	A/Environment/Hong Kong/HKUSPH_MP22_189A/2022	A/Eurasian Wigeon/Hong Kong/AFCD-HKU-23-14524-01009/2023	A/Jiangsu/NJ210/2023
A/Environment/Hong Kong/HKUSPH_MP23_532P1/2023	1.000	1.000	0.996	0.988	0.999	0.975	0.987	0.973	1.000	0.948	1.000	0.969	1.000	0.991
A/Eurasian Teal/Hong Kong/AFCD-HKU-23-14383-02001/2023	1.000	1.000	0.996	0.988	0.999	0.975	0.987	0.973	1.000	0.948	1.000	0.969	1.000	0.991
A/Environment/Hong Kong/HKUSPH_MP24_134P1b/2024	0.996	0.996	1.000	0.986	0.996	0.972	0.985	0.970	0.996	0.946	0.996	0.967	0.996	0.989
A/Black_Faced_Spoonbill/Hong Kong/AFCD-HKU-22-21944-01009/2022	0.988	0.988	0.986	1.000	0.988	0.972	0.990	0.971	0.988	0.947	0.988	0.967	0.988	0.994
A_Eurasian_Teal_HongKong_AFCD-HKU-23-14009-01020_2023	0.999	0.999	0.996	0.988	1.000	0.975	0.987	0.973	0.999	0.948	0.999	0.969	0.999	0.991
A/Curlew/Hong Kong/AFCD-HKU-22-01095.01009/2022	0.975	0.975	0.972	0.972	0.975	1.000	0.971	0.995	0.975	0.955	0.975	0.992	0.975	0.975
A/Black Faced Spoonbill/Hong Kong/AFCD-HKU-22-21429-01012/2022	0.987	0.987	0.985	0.990	0.987	0.971	1.000	0.971	0.987	0.945	0.987	0.967	0.987	0.991
A/Black Faced Spoonbill/Hong Kong/AFCD-HKU-21-17198.01012/2021	0.973	0.973	0.970	0.971	0.973	0.995	0.971	1.000	0.973	0.955	0.973	0.991	0.973	0.974
A/Gallinago Stenura/Hong Kong/AFCD-HKU-23-14383-01014/2023	1.000	1.000	0.996	0.988	0.999	0.975	0.987	0.973	1.000	0.948	1.000	0.969	1.000	0.991
A/Peregrine Falcon/Hong Kong/AFCD-HKU-21-013-27/2021	0.948	0.948	0.946	0.947	0.948	0.955	0.945	0.955	0.948	1.000	0.948	0.951	0.948	0.947
A/Eurasian Wigeon/Hong Kong/AFCD-HKU-23-14484-01001/2023	1.000	1.000	0.996	0.988	0.999	0.975	0.987	0.973	1.000	0.948	1.000	0.969	1.000	0.991
A/Environment/Hong Kong/HKUSPH_MP22_189A/2022	0.969	0.969	0.967	0.967	0.969	0.992	0.967	0.991	0.969	0.951	0.969	1.000	0.969	0.969
A/Eurasian Wigeon/Hong Kong/AFCD-HKU-23-14524-01009/2023	1.000	1.000	0.996	0.988	0.999	0.975	0.987	0.973	1.000	0.948	1.000	0.969	1.000	0.991
A/Jiangsu/NJ210/2023	0.991	0.991	0.989	0.994	0.991	0.975	0.991	0.974	0.991	0.947	0.991	0.969	0.991	1.000

MP	A/Eurasian Teal/Hong Kong/AFCD-HKU-23-14383-02001/2023	A/Gallinago Stenura/Hong Kong/AFCD-HKU-23-14383-01014/2023	A/Curlew/Hong Kong/AFCD-HKU-22-01095.01009/2022	A/Eurasian Wigeon/Hong Kong/AFCD-HKU-23-14524-01009/2023	A/Eurasian Wigeon/Hong Kong/AFCD-HKU-23-14484-01001/2023	A_Eurasian_Teal_HongKong_AFCD-HKU-23-14009-01020_2023	A/Jiangsu/NJ210/2023	A/Black Faced Spoonbill/Hong Kong/AFCD-HKU-22-21429-01012/2022	A/Environment/Hong Kong/HKUSPH_MP24_134P1b/2024	A/Environment/Hong Kong/HKUSPH_MP22_189A/2022	A/Black Faced Spoonbill/Hong Kong/AFCD-HKU-21-17198.01012/2021	A/Environment/Hong Kong/HKUSPH_MP23_532P1/2023	A/Black_Faced_Spoonbill/Hong Kong/AFCD-HKU-22-21944-01009/2022	A/Peregrine Falcon/Hong Kong/AFCD-HKU-21-013-27/2021
A/Eurasian Teal/Hong Kong/AFCD-HKU-23-14383-02001/2023	1.000	1.000	0.986	1.000	1.000	0.998	0.989	0.991	0.998	0.977	0.985	1.000	0.989	0.987
A/Gallinago Stenura/Hong Kong/AFCD-HKU-23-14383-01014/2023	1.000	1.000	0.986	1.000	1.000	0.998	0.989	0.991	0.998	0.977	0.985	1.000	0.989	0.987
A/Curlew/Hong Kong/AFCD-HKU-22-01095.01009/2022	0.986	0.986	1.000	0.986	0.986	0.986	0.985	0.987	0.986	0.985	0.993	0.986	0.985	0.991
A/Eurasian Wigeon/Hong Kong/AFCD-HKU-23-14524-01009/2023	1.000	1.000	0.986	1.000	1.000	0.998	0.989	0.991	0.998	0.977	0.985	1.000	0.989	0.987
A/Eurasian Wigeon/Hong Kong/AFCD-HKU-23-14484-01001/2023	1.000	1.000	0.986	1.000	1.000	0.998	0.989	0.991	0.998	0.977	0.985	1.000	0.989	0.987
A_Eurasian_Teal_HongKong_AFCD-HKU-23-14009-01020_2023	0.998	0.998	0.986	0.998	0.998	1.000	0.989	0.991	0.998	0.977	0.985	0.998	0.989	0.987
A/Jiangsu/NJ210/2023	0.989	0.989	0.985	0.989	0.989	0.989	1.000	0.994	0.989	0.975	0.984	0.989	0.994	0.986
A/Black Faced Spoonbill/Hong Kong/AFCD-HKU-22-21429-01012/2022	0.991	0.991	0.987	0.991	0.991	0.991	0.994	1.000	0.991	0.978	0.986	0.991	0.994	0.988
A/Environment/Hong Kong/HKUSPH_MP24_134P1b/2024	0.998	0.998	0.986	0.998	0.998	0.998	0.989	0.991	1.000	0.977	0.985	0.998	0.989	0.987
A/Environment/Hong Kong/HKUSPH_MP22_189A/2022	0.977	0.977	0.985	0.977	0.977	0.977	0.975	0.978	0.977	1.000	0.984	0.977	0.975	0.982
A/Black Faced Spoonbill/Hong Kong/AFCD-HKU-21-17198.01012/2021	0.985	0.985	0.993	0.985	0.985	0.985	0.984	0.986	0.985	0.984	1.000	0.985	0.984	0.990
A/Environment/Hong Kong/HKUSPH_MP23_532P1/2023	1.000	1.000	0.986	1.000	1.000	0.998	0.989	0.991	0.998	0.977	0.985	1.000	0.989	0.987
A/Black_Faced_Spoonbill/Hong Kong/AFCD-HKU-22-21944-01009/2022	0.989	0.989	0.985	0.989	0.989	0.989	0.994	0.994	0.989	0.975	0.984	0.989	1.000	0.986
A/Peregrine Falcon/Hong Kong/AFCD-HKU-21-013-27/2021	0.987	0.987	0.991	0.987	0.987	0.987	0.986	0.988	0.987	0.982	0.990	0.987	0.986	1.000

NS	A/Peregrine Falcon/Hong Kong/AFCD-HKU-21-013-27/2021	A/Environment/Hong Kong/HKUSPH_MP22_189A/2022	A/Jiangsu/NJ210/2023	A/Eurasian Wigeon/Hong Kong/AFCD-HKU-23-14484-01001/2023	A/Eurasian Wigeon/Hong Kong/AFCD-HKU-23-14524-01009/2023	A/Curlew/Hong Kong/AFCD-HKU-22-01095.01009/2022	A/Eurasian Teal/Hong Kong/AFCD-HKU-23-14383-02001/2023	A/Black Faced Spoonbill/Hong Kong/AFCD-HKU-22-21429-01012/2022	A/Black_Faced_Spoonbill/Hong Kong/AFCD-HKU-22-21944-01009/2022	A/Gallinago Stenura/Hong Kong/AFCD-HKU-23-14383-01014/2023	A/Environment/Hong Kong/HKUSPH_MP23_532P1/2023	A/Environment/Hong Kong/HKUSPH_MP24_134P1b/2024	A_Eurasian_Teal_HongKong_AFCD-HKU-23-14009-01020_2023	A/Black Faced Spoonbill/Hong Kong/AFCD-HKU-21-17198.01012/2021
A/Peregrine Falcon/Hong Kong/AFCD-HKU-21-013-27/2021	1.000	0.916	0.917	0.917	0.917	0.920	0.917	0.919	0.917	0.917	0.916	0.914	0.917	0.922
A/Environment/Hong Kong/HKUSPH_MP22_189A/2022	0.916	1.000	0.941	0.944	0.944	0.981	0.944	0.943	0.941	0.944	0.941	0.943	0.944	0.983
A/Jiangsu/NJ210/2023	0.917	0.941	1.000	0.986	0.986	0.947	0.986	0.994	0.993	0.986	0.983	0.982	0.986	0.950
A/Eurasian Wigeon/Hong Kong/AFCD-HKU-23-14484-01001/2023	0.917	0.944	0.986	1.000	1.000	0.950	1.000	0.987	0.983	1.000	0.998	0.996	0.998	0.952
A/Eurasian Wigeon/Hong Kong/AFCD-HKU-23-14524-01009/2023	0.917	0.944	0.986	1.000	1.000	0.950	1.000	0.987	0.983	1.000	0.998	0.996	0.998	0.952
A/Curlew/Hong Kong/AFCD-HKU-22-01095.01009/2022	0.920	0.981	0.947	0.950	0.950	1.000	0.950	0.949	0.947	0.950	0.947	0.949	0.950	0.990
A/Eurasian Teal/Hong Kong/AFCD-HKU-23-14383-02001/2023	0.917	0.944	0.986	1.000	1.000	0.950	1.000	0.987	0.983	1.000	0.998	0.996	0.998	0.952
A/Black Faced Spoonbill/Hong Kong/AFCD-HKU-22-21429-01012/2022	0.919	0.943	0.994	0.987	0.987	0.949	0.987	1.000	0.992	0.987	0.984	0.983	0.987	0.951
A/Black_Faced_Spoonbill/Hong Kong/AFCD-HKU-22-21944-01009/2022	0.917	0.941	0.993	0.983	0.983	0.947	0.983	0.992	1.000	0.983	0.981	0.980	0.983	0.950
A/Gallinago Stenura/Hong Kong/AFCD-HKU-23-14383-01014/2023	0.917	0.944	0.986	1.000	1.000	0.950	1.000	0.987	0.983	1.000	0.998	0.996	0.998	0.952
A/Environment/Hong Kong/HKUSPH_MP23_532P1/2023	0.916	0.941	0.983	0.998	0.998	0.947	0.998	0.984	0.981	0.998	1.000	0.994	0.995	0.950
A/Environment/Hong Kong/HKUSPH_MP24_134P1b/2024	0.914	0.943	0.982	0.996	0.996	0.949	0.996	0.983	0.980	0.996	0.994	1.000	0.994	0.951
A_Eurasian_Teal_HongKong_AFCD-HKU-23-14009-01020_2023	0.917	0.944	0.986	0.998	0.998	0.950	0.998	0.987	0.983	0.998	0.995	0.994	1.000	0.952
A/Black Faced Spoonbill/Hong Kong/AFCD-HKU-21-17198.01012/2021	0.922	0.983	0.950	0.952	0.952	0.990	0.952	0.951	0.950	0.952	0.950	0.951	0.952	1.000

Table S2: Mammalian adaptation or pathogenesis related mutation

					**Publc samples**			**G7**		**G10**					**G1**				**H5N8**
Gene	Mutation	H5 numbering	H3 numbering	A/Jiangsu/NJ 210/2023	A/wild_duck/Hebei/SD 012/2021	A/Astrakhan/ 3212/2020	A/Environment/Hong Kong/HKUSPH_MP22_189A/2022	**A/Curlew/HongKong/AFCD-HKU-22-01095.01009/2022**	A/Black_Faced_Spoonbill/Hong Kong/AFCD-HKU-21-17198.01012/2021	**A/Black_Faced_Spoonbill/Hong Kong/AFCD-HKU-22-21429-01012/2022**	**A/Black_Faced_Spoonbill/Hong Kong/AFCD-HKU-22-21944-01009/2022**	A/Eurasian_Teal//Hong Kong/AFCD-HKU-23-14383-02001/2023	A/Eurasian_Wigeon/Hong Kong/AFCD-HKU-23-14524-01009/2023	A/Gallinago_Stenura/Hong Kong/AFCD-HKU-23-14383-01014/2023	A/Eurasian_Wigeon/Hong Kong/AFCD-HKU-23-14484-01001/2023	**A/Environment/Hong Kong/HKUSPH_MP23_532P1/2023**	**A/Eurasian_Teal/Hong Kong/AFCD-HKU-23-14009-01020/2023**	A/Environment/Hong Kong/HKUSPH_MP24_134P1b/2024	A/Peregrine_Falcon/Hong Kong/AFCD-HKU-21-013-27/2021
HA	S128P	123	128	P	P	P	P	P	P	P	P	P	P	P	P	P	P	P	P
HA	S137A	133	137	A	A	A	A	A	A	A	A	A	A	A	A	A	A	A	A
HA	N158D	154	158	D	N	N	N	N	N	D	D	N	N	N	N	N	N	N	N
HA	T160A	156	160	A	A	A	A	A	A	A	A	A	A	A	A	A	A	A	A
HA	D187N	183	187	N	N	N	N	N	N	N	N	N	N	N	N	N	N	N	N
HA	K193R/D/N/S	189	193	N	N	N	D	D	D	N	N	N	N	N	N	N	N	N	N
HA	Q196K	192	196	K	K	K	K	K	K	K	K	K	K	K	K	K	K	K	K
HA	T192I	188	192	T	T	T	T	T	T	T	T	T	T	T	T	T	T	T	I
PB2	L89V	89		V	V	V	V	V	V	V	V	V	V	V	V	V	V	V	V
PB2	L89V + G309D	309		D	D	D	D	D	D	D	D	D	D	D	D	D	D	D	D
PB2	I292V	292		A	I	V	I	I	I	V	V	I	M	M	M	I	I	I	V
PB2	K389R	389		R	R	R	R	R	R	R	R	R	R	R	R	R	R	R	R
PB2	V598T	598		T	T	T	T	T	T	T	T	T	T	T	T	T	T	T	T
PB2	K482R	482		K	R	K	K	K	K	K	K	R	R	R	R	R	R	R	K
PA	V63I	63		V	V	V	V	V	V	I	I	V	V	V	V	V	V	V	V
PA	H266R	266		R	R	R	R	R	R	R	R	R	R	R	R	R	R	R	R
PA	F277S	277		S	S	S	S	S	S	S	S	S	S	S	S	S	S	S	S
PA	N383D	383		D	D	D	D	D	D	D	D	D	D	D	D	D	D	D	D
PA	N409S	409		S	S	S	S	S	S	S	S	S	S	S	S	S	S	S	S
PA	S515T	515		T	T	T	T	T	T	T	T	T	T	T	T	T	T	T	T
PA	S37A	37		A	A	A	A	A	A	A	A	A	A	A	A	A	A	A	A
PA	P190S	190		S	S	S	S	S	S	S	S	S	S	S	S	S	S	S	S
PA	N383D	383		D	D	D	D	D	D	D	D	D	D	D	D	D	D	D	D
PA	Q400P	400		P	P	S	S	S	S	P	P	P	P	P	P	P	P	P	S
PB1	D3V	3		V	V	V	V	V	V	V	-	V	V	V	V	V	V	V	V
PB1	D622G	622		G	G	G	G	G	G	G	G	G	G	G	G	G	G	G	G
PB1-F2	N66S	66		S	S	N	N	N	N	S	S	S	S	S	S	S	S	N	N
NP	M105V	105		V	V	V	M	M	M	V	V	V	V	V	V	V	V	V	V
NP	A184K	184		K	K	K	K	K	K	K	K	K	K	K	K	K	K	K	K
NP	N319K	319		N	K	N	N	N	N	N	N	K	K	K	K	K	K	K	N
M1	N30D	30		D	D	D	D	D	D	D	D	D	D	D	D	D	D	D	D
M1	T215A	215		A	A	A	A	A	A	A	A	A	A	A	A	A	A	A	A
M1	I43M	43		M	M	M	M	M	M	M	M	M	M	M	M	M	M	M	M
NS1	P42S	42		S	S	S	S	S	S	S	S	S	S	S	S	S	S	S	S
NS1	I106M	106		M	M	M	M	M	M	M	M	M	M	M	M	M	M	M	M
NS1	C138F	138		F	F	F	F	F	F	F	F	F	F	F	F	F	F	F	F
NS1	V149A	149		A	A	A	A	A	A	A	A	A	A	A	A	A	A	A	A
NS1	L103F + I106M	103		F	F	F	F	F	F	F	F	F	F	F	F	F	F	F	F
NS1	K55E + K66E + C138F	55		E	E	E	D	E	E	E	E	E	E	E	E	E	E	E	E
NS1		66		E	E	K	E	E	E	E	E	E	E	E	E	E	E	E	K

Table S3. Sero-prevalence of hemagglutination inhibition (HAI) and microneutralization (MN) antibodies to Avian influenza A/Black Faced Spoonbill/Hong Kong/AFCD_22-21944.01009/2022(H5N1) in age stratified human sera collected from blood donors in 2020.

Age group	N	H5N1 HAI titre. Number of positive	H5N1 MN titre. Number of positive
		≥1:10	≥1:10
10-19	10	0	0
20-29	10	0	0
30-39	10	0	0
40-49	10	0	0
50-59	10	0	0
60-69	10	0	0
70-79	3	0	0
**Total**	**63**	0	0

Table S4 used strains in Phylogenetic tree

A/bean goose/Germany-BB/AI00444/2021_H5N8
A_Black_faced_Spoonbill_AFCD_22-21429.01012/2022_H5N1
A_chicken_Akita_TU22-31_2022_H5N1
A_chicken_Aomori_TU18-143_144_2022_H5N1
A_chicken_Aomori_TU19-107_108_2022_H5N1
A_chicken_Hokkaido_TU20-28_29_2022_H5N1
A_chicken_Miyagi_TU17-17_18_2022_H5N1
A_Eurasianwigeon_SouthKorea_23WS022-22_2023_H5N1
A_junglecrow_Hokkaido_0104B085_2022_H5N1
A_junglecrow_Hokkaido_0104B087_2022_H5N1
A_junglecrow_Iwate_0303I003_2022_H5N1
A_Northern_Pintail_USA_IZ22_0335_swab_2022_H5N1
A_pelican_Tumen_1032-1_2021_H5N1
A_pelican_Tumen_932-1_2021_H5N1
A_white-frontedgoose_Iwate_TU16-74_2022_H5N1
A_whooperswan_Iwate_0302I001T_2022_H5N1
A_whooperswan_Iwate_0303B006_2022_H5N1
A_Wild_duck_South_Korea_KNU2020-58_2020
A/American Wigeon/USA/IZ22_0890/2022_H5N1
A/Anas platyrhynchos/Belgium/204_0003/2020_H4N6
A/Anas platyrhynchos/Belgium/9594H191810/2016_H1N1
A/Anas_platyrhynchos/Belgium/10402_H195386/2017_H1N1
A/Anas_platyrhynchos/Belgium/827/2020_H3N8
A/Anser_albifrons/Belgium/15465_0010/2021_H5N1
A/anser_anser/Spain/297-1_21VIR1230-5/2021_H5N8
A/Anser_brachyrhynchus/Belgium/13275_0009/2020_H5N8
A/Anser_brachyrhynchus/Belgium/151/2020_H5N8
A/Astrakhan/3212/2020_H5N8
A/Aviafauna/Kazakhstan/2020_H5N8
A/baldeagle/Alaska/22-013831-001/2022_H5N1
A/bar_headed_goose/Tibet/T1640/2021_H5N8
A/bar_headed_goose/Tibet/T1707/2021_H5N8
A/Bar-headedGoose/Tibet/XZ71/2021_H5N8
A/Bar-headedGoose/Tibet/XZ81/2021_H5N8
A/Bar-headedGoose/Tibet/XZQ10-1/2021_H5N8
A/Bar-headedGoose/Tibet/XZQ5-1/2021_H5N8
A/Barnacle_goose/Scotland/003169/2021_H5N1
A/Barnacle_goose/Scotland/003579/2022_H5N1
A/Barnacle_goose/Scotland/072140/2021_H5N1
A/Barnacle_goose/Scotland/072152/2021_H5N1
A/barnacle_goose/Sweden/SVA210225SZ0307/KN000666/2021_H5N8
A/barnaclegoose/Germany-SH/AI02167/2020_H5N8
A/barnaclegoose/Germany-SH/AI02168/2020_H5N8
A/barnaclegoose/Germany-SH/AI02190/2020_H5N8
A/barnaclegoose/Germany-SH/AI02379/2020_H5N8
A/barnaclegoose/Netherlands/21038248-001/2021_H5N1
A/Barnaclegoose/Netherlands/3/2022_H5N1

A/barnaclegoose/Sweden/SVA201201SZ0353/KN003624/2020_H5N5
A/barnaclegoose/Sweden/SVA201215SZ0368/KN003768/2020_H5N8
A/barnaclegoose/Sweden/SVA210126SZ0462/KN000202/2021_H5N8
A/barnaclegoose/Sweden/SVA210202SZ0453/KN000276/2021_H5N8
A/barnaclegoose/Sweden/SVA210210SZ0372/FB000374/M-2021_H5N8
A/barnaclegoose/Sweden/SVA210423SZ0252/FB001647/M2021_H5N8
A/beangoose/Hubei/BQ11/2020_H5N8
A/blowfly/Kagoshima/23a738D/2023_H5N1
A/Brown-headedGull/Tibet/XZQ15-2/2021_H5N8
A/Buteo_buteo/Belgium/00331-0007/2022_H5N1
A/Canada_goose/Sweden/SVA210302SZ0455/KN000714/SKsim/2021_H5N8
A/Canadagoose/Sweden/SVA210209SZ0420/KN000345/2021_H5N8
A/Canadagoose/Sweden/SVA210305SZ0255/KN000797/SK/2021_H5N8
A/Canadagoose/Sweden/SVA210331SZ/FB001326/W-2021_H5N8
A/Canadagoose/Sweden/SVA210407SZ0510/FB001379/O-2021_H5N8
A/chicken/Akita/TU22-31/2022_H5N1
A/chicken/Aomori/TU18-143/144/2022/H5N1
A/chicken/Aomori/TU18-143144/2022_H5N1
A/chicken/Aomori/TU19-107/108/2022/H5N1
A/chicken/Aomori/TU19-107108/2022_H5N1
A/chicken/Aomori/TU9-41,42/2021_H5N1
A/chicken/Astrakhan/321-09/2020_H5N8
A/chicken/Chiba/E3T/2021_H5N8
A/chicken/Chiba/F3C/2021_H5N8
A/chicken/Chiba/G1C/2021_H5N8
A/chicken/Chiba/G1T/2021_H5N8
A/chicken/Chiba/G2C/2021_H5N8
A/chicken/Chiba/G2T/2021_H5N8
A/chicken/Chiba/H1C/2021_H5N8
A/chicken/Chiba/H1T/2021_H5N8
A/chicken/Chiba/I1C/2021_H5N8
A/chicken/Chiba/I2T/2021_H5N8
A/chicken/Chiba/I3T/2021_H5N8
A/chicken/Chiba/K5T/2021_H5N8
A/chicken/Chiba/T46-11/2021_H5N8
A/chicken/Chiba/T48-17/2021_H5N8
A/chicken/Chiba/T49-18/2021_H5N8
A/chicken/Chiba/T50-23/2021_H5N8
A/chicken/Chiba/TU14-1_2/2022_H5N1
A/chicken/Chiba/TU14-12/2022_H5N1
A/chicken/China/JM01/2020_H5N8
A/chicken/Egypt/F17230B/2019_H5N8
A/chicken/Egypt/N16732/2019_H5N8
A/chicken/Ehime/TU10-2-13/2022_H5N1
A/chicken/Ehime/TU11-2-24,25/2022_H5N1
A/chicken/Ehime/TU12-2-16,17/2022_H5N1
A/chicken/England/007584/2022_H5N1
A/chicken/England/072926/2021_H5N1

A/chicken/France/21P007258/2021_H5N8
A/chicken/France/21P008169/2021_H5N8
A/chicken/Germany-BE/AI01974/2021_H5N8
A/chicken/Hokkaido/TU20-28/29/2022/H5N1
A/chicken/Hokkaido/TU20-2829/2022_H5N1
A/chicken/Hokkaido/TU25-3/2022_H5N1
A/chicken/Hyogo/TU4-45,46/2021_H5N1
A/chicken/Italy/21VIR10352/2021_H5N1
A/chicken/Japan/TU16-1112/2022_H5N1
A/chicken/Jiangxi/S40653/2021_H5N1
A/chicken/Kagoshima/21A6T/2021_H5N1
A/chicken/Kagoshima/TU13-3_4/2022_H5N1
A/chicken/Kagoshima/TU13-34/2022_H5N1
A/chicken/Kagoshima/TU2-18,19/2021_H5N1
A/chicken/Kagoshima/TU3-46,47/2021_H5N8
A/chicken/Kazakhstan/1-20-B-Talg-67/2020_H5N8
A/chicken/Kazakhstan/12-20-B-Talg-45/2020_H5N8
A/chicken/Kazakhstan/23/2020_H5N8
A/chicken/Khabarovsk/24-10V/2022_H5N1
A/chicken/Khabarovsk/24-12V/2022_H5N1
A/chicken/Khabarovsk/24-3V/2022_H5N1
A/chicken/Kostroma/304-01/2020_H5N8
A/chicken/Kostroma/304-04/2020_H5N8
A/chicken/Kostroma/304-06/2020_H5N8
A/chicken/Kostroma/304-08/2020_H5N8
A/chicken/Krasnodar/334-01/2021_H5N8
A/chicken/Kurgan/1004/2020_H5N8
A/chicken/Lesotho/341.10/2021_H5N1
A/chicken/Miyagi/TU17-1718/2022_H5N1
A/chicken/Miyazaki/J3T/2021_H5N8
A/chicken/Netherlands/21038165-006010/2021_H5N1
A/chicken/Netherlands/21039901-001-005/2021_H5N1
A/chicken/Nigeria/VRD21-035B_21VIR2288-1/2021_H5N8
A/chicken/Nigeria/VRD21-035B/21VIR2288-1/2021_H5N8
A/chicken/Nigeria/VRD21-102_21VIR2370-424/2021_H5N1
A/chicken/Nigeria/VRD21-109_21VIR2370-425/2021_H5N1
A/chicken/Nigeria/VRD21-109/21VIR2370-425/2021_H5N1
A/chicken/Nigeria/VRD21-37_21VIR2288-2/2021_H5N1
A/chicken/Nigeria/VRD21-43_21VIR2288-4/2021_H5N8
A/chicken/Nigeria/VRD21-53B_21VIR2288-5/2021_H5N8
A/chicken/Nigeria/VRD21-98_21VIR2288-6/2021_H5N1
A/chicken/Omsk/0112/2020_H5N8
A/chicken/Omsk/0118/2020_H5N8
A/chicken/Omsk/0119/2020_H5N8
A/chicken/Rostov-on-Don/159-1V/2021_H5N1
A/chicken/Saitama/TU7-34,36/2021_H5N1
A/chicken/Serbia/22337-21/2021_H5N1
A/chicken/Tokushima/B1T/2020_H5N8

A/chicken/Tokushima/B3T/2020_H5N8
A/chicken/Tottori/TU22-17/2022_H5N1
A/chicken/Tyumen/27-40V/2021_H5N1
A/chicken/Tyumen/47-79V/2021_H5N1
A/chicken/Tyumen/81-97V/2021_H5N1
A/chicken/Vietnam/HU14-LB11/2021_H5N8
A/chicken/Vietnam/NCVD-15A59/2015_H5N6
A/chicken/Vietnam/Raho4-Cd-20-421/2020_H5N6
A/Chlidonias hybrida/Hubei/55/2020_H5N8
A/Chlidoniashybrida/Hubei/55/2020_H5N8
A/common buzzard/Japan/2601B013/2022_H5N1
A/common buzzard/Kyoto/2601B013/2022_H5N1
A/common eiders/Maine/W22-481B/2022_H5N1
A/common pochard/Egypt/DT19799C/2021_H5N8
A/Common Teal/Amur region/31b/2019_H3N6
A/common teal/Chelyabinsk/1379-1/2021_H5N1
A/common tern/Maine/W22-480B/2022_H5N1
A/common_buzzard/England/245061/2021_H5N1
A/common_gull/England/245481/2022_H5N1
A/common_teal/Chelyabinsk/1379-1/2021_H5N1
A/commonbuzzard/Kyoto/2601B013/2022/H5N1
A/commonbuzzard/Netherlands/21038793-001/2021_H5N1
A/commoncrane/Yunnan-Huize/27/2021(H5N8)_H5N8
A/commoneiders/Maine/W22-481B/2022_H5N1
A/commonteal/Chelyabinsk/1379-1/2021/H5N1
A/commonteal/Chiba/T42-F1/2021_H5N8
A/crow/Fukuoka/TU54-47/2023/H5N1
A/crow/Hokkaido/0101Q056/2022_H5N1
A/crow/Hokkaido/0102F043/2022_H5N1
A/crow/Hokkaido/0102F046/2022_H5N1
A/crow/Hokkaido/0102L015/2022_H5N1
A/crow/Hokkaido/0103B065/2022_H5N1
A/crow/Hokkaido/0103L018/2022_H5N1
A/crow/Japan/TU-06/2022_H5N1
A/crow/Japan/TU-19/2023_H5N1
A/crow/Japan/TU-22/2022_H5N1
A/crow/Khabarovsk/776-56/2022_H5N1
A/Cygnus columbianus/Hubei/50/2020_H5N8
A/Cygnus columbianus/Hubei/51/2020_H5N8
A/Cygnus columbianus/Hubei/52/2020_H5N8
A/Cygnuscolumbianus/Hubei/116/2020_H5N8
A/Cygnuscolumbianus/Hubei/117/2021_H5N1
A/Cygnuscolumbianus/Hubei/121/2021_H5N1
A/Cygnuscolumbianus/Hubei/123/2021_H5N1
A/Cygnuscolumbianus/Hubei/126/2021_H5N1
A/Cygnuscolumbianus/Hubei/127/2021_H5N1
A/Cygnuscolumbianus/Hubei/128/2021_H5N1
A/Cygnuscolumbianus/Hubei/49/2020_H5N8

A/Cygnuscolumbianus/Hubei/50/2020_H5N8
A/Cygnuscolumbianus/Hubei/51/2020_H5N8
A/Cygnuscolumbianus/Hubei/52/2020_H5N8
A/Cygnuscolumbianus/Hubei/53/2020_H5N8
A/Cygnuscolumbianus/Hubei/56/2020_H5N8
A/domestic_duck/England/017166/2022_H5N1
A/domestic_duck/England/058612/2021_H5N1
A/domestic_duck/England/073865/2022_H5N1
A/domesticduck/Chiba/T37-1-2/2021_H5N8
A/domesticduck/Germany-ST/AI03183/2021_H5N8
A/duck/Bangladesh/19D1874/2022_H5N1
A/duck/Bangladesh/51600/2021_H5N1
A/duck/Bangladesh/51601/2021_H5N1
A/duck/Bangladesh/51602/2021_H5N1
A/duck/Chiba/C5T/2021_H5N8
A/duck/Chiba/D1A-9T/2021_H5N8
A/duck/Chiba/D1B-2T/2021_H5N8
A/duck/Chiba/D1B-5T/2021_H5N8
A/duck/Chiba/D2A-5T/2021_H5N8
A/duck/Chiba/D2B-9C/2021_H5N8
A/duck/Chiba/TU6-19,20/2021_H5N1
A/duck/Egypt/A16368/2018_H5N8
A/duck/Egypt/A16793/2019_H5N8
A/duck/Egypt/BA19903OP/2021_H5N8
A/duck/Egypt/BA20360C/2022_H5N1
A/duck/France/21343/2021_H5N1
A/duck/Guangdong/S4518/2021_H5N1
A/duck/Guangdong/S4525/2021_H5N1
A/duck/Guizhou/S1321/2022_H5N1
A/duck/Hubei/SE128/2022_H5N1
A/duck/Hubei/SE220/2022_H5N1
A/duck/Hulu Sungai Utara/A0522064-03-04/2022_H5N1
A/duck/Hulu Sungai Utara/A0522064-06/2022_H5N1
A/duck/Hyogo/1/2016_H5N6
A/duck/Kazakhstan/12-20-B-Talg-11/2020_H5N8
A/duck/Korea/H125/2022_H5N1
A/duck/Korea/H493/2022_H5N1
A/duck/Mongolia/826/2019_H4N6
A/duck/Moscow/5586/2018_H1N2
A/duck/NorthernChina/LSP/2020(H5N8)_H5N8
A/duck/NorthernChina/ZGL/2020(H5N8)_H5N8
A/duck/Shandong/SD0261/2021_H5N6
A/duck/SouthwesternChina/B1904/2020(H5N8)_H5N8
A/duck/Tottori/NK40/2023_H5N1
A/duck/Tottori/TG164/2023_H5N1
A/Duck/Vientiane/987/2022_H5N1
A/duck/Zhejiang/S4854/2021_H5N6
A/easternbuzzard/Tochigi/0901B015/2023_H5N1

A/egret/France/21P013418/2021_H5N1
A/emu/Hokkaido/A-2/2022_H5N1
A/emu/Hokkaido/TU21-1_2/2022_H5N1
A/emu/Iwate/TU24-19_20/2022_H5N1
A/emu/Iwate/TU24-1920/2022_H5N1
A/emu/Japan/TU21-12/2022_H5N1
A/environment sample/China/TZ001/2021_H5N8
A/environment/Akita/TU1-49/2021_H5N8
A/Environment/Guangdong/14452/2018_H3N2
A/environment/sample/China/TZ001/2021_H5N8
A/environment/Tokushima/T47-36/2021_H5N8
A/environmentsample/China/TZ001/2021_H5N8
A/eurasian coot/Shandong/W5611/2020_H5N8
A/Eurasian wigeon/Netherlands/1/2020_H5N1
A/eurasiancoot/Shandong/W5611/2020_H5N8
A/eurasiancoot/Shandong/W6143/2020_H5N8
A/Eurasianwigeon/Hokkaido/M184/2022_H5N1
A/Eurasianwigeon/Hokkaido/Q71/2022_H5N1
A/falcatedteal/Shanghai/JDS20857/2020_H5N8
A/falcon/England/AVP-21-019051A/2021_H5N8
A/Fujian-Sanyuan/21099/2017_H5N6
A/gadwall/Chany/893/2018_H3N8
A/glaucous gull/USA/22-031916-001/1905_H5N1
A/goose/Chelyabinsk/1341-3/2021_H5N1
A/goose/China/1/2021_H5N8
A/goose/China/21FU003/2020_H5N8
A/goose/China/21FU005/2020_H5N8
A/goose/France/21P014207/2021_H5N1
A/goose/Guizhou/S1541/2022_H5N1
A/goose/Hebei/HG12/2021_H5N8
A/goose/Hunan/SE284/2022_H5N1
A/goose/Magadan/2272-5/2022_H5N1
A/goose/Netherlands/21038940-002/2021_H5N1
A/goose/Netherlands/21039029-001/2021_H5N1
A/goose/Omsk/0111/2020_H5N8
A/goose/Omsk/011101/2020_H5N8
A/goose/Omsk/0113/2020_H5N8
A/goose/Omsk/01171/2020_H5N8
A/goose/Omsk/30009/2020_H5N8
A/goshawk/Gifu/1/2023_H5N1
A/green sandpiper/Kurgan/1050/2018_H3N8
A/green-winged teal/USA/IZ22_0887/2022_H5N1
A/greyheron/Czech_Republic/23608/2021_H5N1
A/greylag_goose/Northern_Ireland/17795/2021_H5N1
A/greylaggoose/Germany-SH/AI06144/2021_H5N1
A/Guangdong/1/2021_H5N6
A/Guangdong/18SF020/2018_H5N6
A/Guangdong/lgf/2021_H5N6

A/GX-guilin/11151/2021_H5N6
A/gyrfalcon/Washington/41088-6/2014_H5N8
A/Hangzhou/01/2021_H5N6
A/Harris'shawk/Hyogo/HU-FA003/2023_H5N1
A/Harriss hawk/Hyogo/HU-FA003/2023_H5N1
A/Hubei/29578/2016_H5N6
A/ibis/Egypt/RLQP-229S/2022_H5N1
A/Jiangsu/NJ210/2023_H5N1
A/jungle crow/Akita/0504F001/2022_H5N1
A/jungle crow/Hokkaido/0104B087/2022_H5N1
A/jungle crow/Iwate/0304I001/2022_H5N1
A/jungle crow/Japan/0303I003/2022_H5N1
A/junglecrow/Japan/0104B085/2022_H5N1
A/junglecrow/Japan/0104B087/2022_H5N1
A/junglecrow/Japan/0504F001/2022_H5N1
A/large-billedcrow/Hokkaido/4810Z002C/2023_H5N1
A/large-billedcrow/Hokkaido/B067/2023_H5N1
A/large-billedcrow/Hokkaido/B068/2023_H5N1
A/large-billedcrow/Osaka/HU-FA004/2023_H5N1
A/large-billedcrow/Yamagata/0601A009/2023_H5N1
A/mallard duck/Tennessee/TN22-303/2022_H5N1
A/mallard/Denmark/12947-12/2020/H7N5
A/mallard/Miyazaki/4501C605/2021_H5N8
A/mallard/Miyazaki/4501C607-c1/2021_H5N8
A/mallard/Miyazaki/4501C607-c3/2021_H5N8
A/mallard/Miyazaki/4501C607-c5/2021_H5N8
A/mallard/Miyazaki/4501C607-c7/2021_H5N8
A/mallard/Netherlands/21038796-002/2021_H5N1
A/mallard/Novosibirsk region/1894k/2019_H4N6
A/mallard/Shanghai/JDS20876/2020_H5N8
A/mallard/Shizuoka/2212D006/2022_H5N1
A/mandarinduck/Heilongjiang/HL-1/2021_H5N1
A/mandarinduck/Heilongjiang/HL-2/2021_H5N1
A/Mandarinduck/Korea/WA496/2022_H5N1
A/mandarinduck/Korea/WA585/2021_H5N1
A/Mule_duck/France/20339/2020_H5N8
A/Mule_duck/France/20347/2020_H5N8
A/Muscovyduck/Vietnam/HN5901/2019_H4N6
A/mute_swan/Northern_Ireland/17404/2021_H5N1
A/mute_swan/Northern_Ireland/17634/2021_H5N1
A/muteswan/Croatia/100/2021_H5N1
A/muteswan/Ibaraki/080203C/2021_H5N8
A/muteswan/Ibaraki/080203T/2021_H5N8
A/muteswan/Ibaraki/0812M001/2022_H5N1
A/northern pintail/USA/IZ22_0783/2022_H5N1
A/northern pintail/USA/IZ22_0812/2022_H5N1
A/northernpintail/Alaska/IZ22/0335/2022/H5N1
A/northernpintail/Shanghai/JDS20843/2020_H5N8

A/pelican/Tumen/1032-1/2021_H5N1
A/peregrinefalcon/Kanagawa/1409C001T1/2022_H5N1
A/peregrinefalcon/Miyagi/0412B001/2022_H5N1
A/Phalacrocorax_carbo/Belgium/1734_0002/2022_H5N1
A/pigeon/Germany-NW/AI00951/2022_H5N1
A/pigeon/Kazakhstan/15-20-B-Talg-5/2020_H5N8
A/poultry/Benin/21-A-08-035-O/2021_H5N1
A/poultry/Benin/21-A-09-031-O/2021_H5N1
A/quail/Korea/H526/2021_H5N1
A/red fox/USA/22-016300-001/2022_H5N1
A/red-crownedcrane/Hokkaido/20231114001/2023_H5N1
A/Sichuan/26221/2014_H5N6
A/spot-billed duck/Shanghai/JDS20867/2020_H5N8
A/Spot-billed_duck/Korea/K22-730-1/2022_H5N1
A/Spot-billed_duck/Korea/K22-856-2/2022_H5N1
A/Spot-billed_duck/Korea/K22-862-1/2022_H5N1
A/Spot-billed_duck/Korea/K22-920/2022_H5N1
A/spot-billedduck/Gifu/1118/2023_H5N1
A/Spot-billedDuck/Ningxia/Y16/2021_H5N8
A/Spot-billedDuck/Ningxia/Y26/2021_H5N8
A/swan/France/21P006202/2021_H5N8
A/swan/Kazakhstan/1-267-20-B-Talg-52/2020_H5N8
A/swan/Romania/10986_22VIR2749-8/2022_H5N1
A/swan/Serbia/19524-21/2021_H5N1
A/teal/Miyazaki/211109-32/2021_H5N1
A/tundraswan/Shanghai/CM20111601/2020_H5N8
A/turkey/England/009687/2022_H5N1
A/turkey/England/038115/2020_H5N8
A/turkey/Germany-BB/AI02434/2021_H5N8
A/turkey/Israel/564/2021_H5N1
A/turkey/Italy/21VIR8585-1/2021_H5N1
A/turkey/Italy/21VIR9520-3/2021_H5N1
A/turkey/Netherlands/21040980-001005/2021_H5N1
A/turkey/Omsk/0003/2020_H5N8
A/turkey/Rostov-on-Don/332-10/2021_H5N8
A/Turkey/Sweden/SVA201118SZ0002/KN305633-IP1/2020_H5N8
A/turkey/Tyumen/15-1V/2021_H5N1
A/turkey/Tyumen/81-96V/2021_H5N1
A/turkey/Wales/065047/2021_H5N1
A/water/Nagano/SW0214/2021_H5N8
A/whiskeredtern/Hubei/BQ10/2020_H5N8
A/white_stork/France/21P007383/2021_H5N8
A/white-fronted goose/Iwate/TU16-74/2022_H5N1
A/white-frontedgoose/Japan/TU16-74/2022_H5N1
A/white-tailed_eagle/Japan/OU-1/2022_H5N1
A/white-tailedeagle/Hokkaido/20220210001/2022_H5N1
A/whooper swan/Iwate/0302I001T/2022_H5N1
A/whooper swan/Iwate/0303B006/2022_H5N1

A/whooper swan/Shanxi/4-1/2020_H5N8
A/whooperswan/Akita/0511B002/2022_H5N1
A/whooperswan/Gunma/1012A011/2022_H5N1
A/whooperswan/Henan/2/2021_H5N1
A/whooperswan/Henan/424/2021_H5N1
A/whooperswan/Henan/6/2021_H5N1
A/whooperswan/Henan/SM61/2020_H5N8
A/whooperswan/Henan/SMQ10/2020_H5N8
A/whooperswan/InnerMongolia/w1-1/2020_H5N8
A/Whooperswan/Sanmenxia/G15/2020_H5N8
A/Whooperswan/Sanmenxia/G2/2020_H5N8
A/Whooperswan/Sanmenxia/H615/2020_H5N8
A/Whooperswan/Sanmenxia/H810/2020_H5N8
A/Whooperswan/Sanmenxia/Y11/2020_H5N8
A/Whooperswan/Sanmenxia/Y12/2020_H5N8
A/Whooperswan/Sanmenxia/Y16/2020_H5N8
A/Whooperswan/Sanmenxia/Y20/2020_H5N8
A/Whooperswan/Sanmenxia/Y26/2020_H5N8
A/Whooperswan/Sanmenxia/Y48B/2020_H5N8
A/Whooperswan/Sanmenxia/Y49/2020_H5N8
A/Whooperswan/Sanmenxia/Y54/2020_H5N8
A/Whooperswan/Sanmenxia/Y57/2020_H5N8
A/Whooperswan/Sanmenxia/Y8/2020_H5N8
A/whooperswan/Shaanxi/SXY2-1/2020_H5N8
A/whooperswan/Shanxi/14/2021_H5N1
A/whooperswan/Shanxi/4-1/2020_H5N8
A/whooperswan/Shanxi/SX206/2020_H5N8
A/whooperswan/Shanxi/SX346/2020_H5N8
A/whooperswan/Tochigi/090203C/2021_H5N8
A/Wild_bird/Korea/K22-742/2022_H5N1
A/wild/duck/Hebei/SD012/2021(H5N1)_H5N1
A/Wildbird/China/Cixi02/2020_H5N8
A/wildduck/Hebei/SD012/2021_H5N1
A/WildDuck/Ningxia/Y54/2021_H5N8
A/wildduck/Omsk/01111/2020_H5N8
A/wildduck/Shandong/W3580/2020_H5N8
A/Dunlin/BC/AIVPHL-2052/2024
A/Merlin/BC/AIVPHL-2103/2024
A/large-billed_crow/Hokkaido/B067/2023
A/Fujian-Sanyuan/21099/2017
A/chicken/Philippines/BA-PTY/2022_H5N1
A/chicken/Philippines/BA-MHN/2022_H5N1
A/dairy_cow/Texas/24-008749-003/2024_H5N1
A/dairy_cow/Texas/24-008749-007/2024_H5N1
A/duck/Hulu_Sungai_Utara/A0522064-06/2022_H5N1
A/turkey/Tyumen/81-96V/2021_H5N1
A/duck/Chiba/22A6T/2022_H5N1
A/chicken/Miyazaki/22B7T/2022_H5N1

A/Env/Changsha/6-3/2022_H5N1
